# Non-invasive intravoxel incoherent motion MRI in prediction of histopathological response to neoadjuvant chemotherapy and survival outcome in osteosarcoma at the time of diagnosis

**DOI:** 10.1186/s12967-022-03838-1

**Published:** 2022-12-27

**Authors:** Esha Baidya Kayal, Sameer Bakhshi, Devasenathipathy Kandasamy, Mehar Chand Sharma, Shah Alam Khan, Venkatesan Sampath Kumar, Kedar Khare, Raju Sharma, Amit Mehndiratta

**Affiliations:** 1 Centre for Biomedical Engineering, Indian Institute of Technology Delhi, Hauz Khas, New Delhi, 110016 India; 2 Department of Medical Oncology, Dr. B.R. Ambedkar Institute-Rotary Cancer Hospital (IRCH), All India Institute of Medical Sciences, New Delhi, India; 3 Department of Radiodiagnosis, All India Institute of Medical Sciences, New Delhi, India; 4 Department of Pathology, All India Institute of Medical Sciences, New Delhi, India; 5 Department of Orthopaedics, All India Institute of Medical Sciences, New Delhi, India; 6 Department of Physics, Indian Institute of Technology Delhi, New Delhi, India; 7 Department of Biomedical Engineering, All India Institute of Medical Sciences, New Delhi, India

**Keywords:** Intravoxel incoherent motion, Diffusion weighted imaging, Biomarkers, Chemotherapy response evaluation, Survival outcome, Osteosarcoma

## Abstract

**Background:**

Early prediction of response to neoadjuvant chemotherapy (NACT) is important to aid personalized treatment in osteosarcoma. Diffusion-weighted Intravoxel Incoherent Motion (IVIM) MRI was used to evaluate the predictive value for response to NACT and survival outcome in osteosarcoma.

**Methods:**

Total fifty-five patients with biopsy-proven osteosarcoma were recruited prospectively, among them 35 patients were further analysed. Patients underwent 3 cycles of NACT (Cisplatin + Doxorubicin) followed by surgery and response adapted adjuvant chemotherapy. Treatment outcomes were histopathological response to NACT (good-response ≥ 50% necrosis and poor-response < 50% necrosis) and survival outcome (event-free survival (EFS) and overall survival (OS)). IVIM MRI was acquired at 1.5T at baseline (t0), after 1-cycle (t1) and after 3-cycles (t2) of NACT. Quantitative IVIM parameters (D, D*, f & D*.f) were estimated using advanced state-of-the-art spatial penalty based IVIM analysis method bi-exponential model with total-variation penalty function (BETV) at 3 time-points and histogram analysis was performed.

**Results:**

Good-responders: Poor-responders ratio was 13 (37%):22 (63%). EFS and OS were 31% and 69% with 16.27 and 25.9 months of median duration respectively. For predicting poor-response to NACT, IVIM parameters showed AUC = 0.87, Sensitivity = 86%, Specificity = 77% at t0, and AUC = 0.96, Sensitivity = 86%, Specificity = 100% at t1. Multivariate Cox regression analysis showed smaller tumour volume (HR = 1.002, *p* = 0.001) higher *ADC*-25^th^-percentile (HR = 0.047, *p* = 0.005) & *D*-Mean (HR = 0.1, *p* = 0.023) and lower D*-Mean (HR = 1.052, *p* = 0.039) were independent predictors of longer EFS (log-rank *p*-values: 0.054, 0.0034, 0.0017, 0.0019 respectively) and non-metastatic disease (HR = 4.33, *p* < 10^–3^), smaller tumour-volume (HR = 1.001, *p* = 0.042), lower D*-Mean (HR = 1.045, *p* = 0.056) and higher D*.f-skewness (HR = 0.544, *p* = 0.048) were independent predictors of longer OS (log-rank *p*-values: < 10^–3^, 0.07, < 10^–3^, 0.019 respectively).

**Conclusion:**

IVIM parameters obtained with a 1.5T scanner along with novel BETV method and their histogram analysis indicating tumour heterogeneity were informative in characterizing NACT response and survival outcome in osteosarcoma.

**Supplementary Information:**

The online version contains supplementary material available at 10.1186/s12967-022-03838-1.

## Background

Neoadjuvant and adjuvant chemotherapy along with surgery have significantly improved the long-term disease-free survival rate of patients with osteosarcoma [1]. Five-year disease-free survival rate for localised osteosarcoma is reported as 60–70%, whereas it is less than 20% in patients presenting with distance metastasis [1], although the survival is relatively lower in resource challenged countries [2, 3]. Measurement of necrosis by histopathological evaluation (HPE) of resected lesion is the current gold standard for neoadjuvant chemotherapy (NACT) response assessment and an important prognostic factor for survival in patients with localized osteosarcoma [4]; however, it is only possible after surgery on completion of NACT. There is no robust, non-invasive and early assessment tool for evaluation of prognosis. Early evaluation of treatment response and prediction of survival outcome may help to prevent the patients from undergoing ineffective chemotherapy regimen and triage them to alternate therapeutic options saving time, cost and side-effects [4–7]. Qualitative evaluation of morphological MR images for intensity variation and tumour size changes, for non-invasive NACT response evaluation in solid tumours, do not always correlate well with the HPE [8, 9]. Therefore, the development of non-invasive radiomics based strategies to select patients with poor prognosis who might benefit from alternative therapy is desirable [10]. 

Quantitative analysis of advanced functional MRI techniques like Diffusion weighted MRI (DWI) [11–17], Perfusion MRI [18, 19] and FDG-PET/CT [13, 20–24] have shown promising results for non-invasive evaluation of NACT response and treatment outcome in osteosarcoma and other tumours. DWI with the associated apparent diffusion coefficient (*ADC*) is a well-established technique for NACT response and outcome evaluation in osteosarcoma, where increase in *ADC* has been shown to indicate good prognosis [11–17]. Using contrast enhanced perfusion MRI, association of hyper-perfusion and hyper-vascularisation of osteosarcoma with poor treatment outcome have been also described [13, 18, 19, 25, 26]. However, DWI has the advantage over perfusion MRI and FDG-PET/CT as it does not involve exogenous contrast agents and might possibly detect treatment effects on tissue microstructure early in the course of NACT. Moreover, Intravoxel Incoherent Motion (IVIM) [27] DWI, separating microcirculatory perfusion from tissue diffusion at lower diffusion-weighting-factors (b-value ≤ 200 s/mm^2^), have shown evidence for prognostic information for various tumours [28–36] along with osteosarcoma [37, 38]. Quantitative IVIM diffusion and perfusion parameters reflect tumor cellularity and vascularity respectively. Since cellular death and vascular changes both occur in response to chemotherapy in lesion, separating diffusion and perfusion component in DWI signal can be more useful in better diagnosis and assessing early response to therapy. However, quantitative IVIM analysis has not been included in routine clinical practice due to poor signal-to-noisy ratio.

Recently developed spatial penalty based IVIM analysis method Biexponential model with Total variation penalty function (BE + TV or BETV) [44] has shown qualitatively and quantitatively improved parameter estimation compared to the commonly used IVIM analysis methods like biexponential model [27] and its segmented variants [28–36]. Robustness of this novel, BETV method has already been demonstrated in cancer simulations & various clinical applications such as, osteosarcoma [39], Ewing sarcoma [40], lymphoma [41], brain tumour [42] and prostate [43] for characterizing tumour and measuring treatment response. Existing studies on DWI performing prediction of NACT response and treatment outcome in osteosarcoma have shown inconclusive or contradictory findings [11] and there is scope for further research. Therefore, this study evaluates the applicability of this state-of-the-art, BETV, IVIM analysis method for oncological applications using clinical datasets of osteosarcoma.

Further, histogram analysis assessing heterogeneity in tumour microenvironment has shown significant improvement in tumour characterisation and predicting the therapeutic response with a more direct correlation with the underlying structural and pathophysiological changes manifested upon tumour progression [44]. Novelty of this study lies in exploring the role of quantitative IVIM analysis for predicting long-term survival outcome in patients with osteosarcoma that has not yet been performed. In this prospective study, assessment of quantitative IVIM diffusion and perfusion parameters and their histogram analysis has been performed to identify potential imaging biomarkers for predicting histopathological response to chemotherapy in tumour and long-term survival outcome after treatment in patients with osteosarcoma before or early in the course of NACT treatment.

## Materials and methods

### Patient population, treatment and follow-up

Patients were enrolled prospectively from March 2016 to March 2018 at Department of Medical Oncology, Dr. B.R.A Institute Rotary Cancer Hospital, All India Institute of Medical Sciences New Delhi, India. Inclusion criteria were treatment naïve patients with biopsy proven osteosarcoma and more than 8 years of age who were planned for NACT. Exclusion criteria were recurrent disease and contradiction to MRI or requiring general anaesthesia for MRI acquisition. All patients underwent NACT, consisting of 3 cycles of Cisplatin and Doxorubicin [45] every 3 weeks. Patients underwent MRI for evaluation of the primary tumour site and chest CT and bone scans for metastatic work-up. After completing three NACT cycles, patients underwent surgery within 3–4 weeks and histological responses to NACT were evaluated on the postsurgical specimens. After surgery all patients underwent 3–6 cycles of adjuvant chemotherapy based on our inhouse protocol with cisplatin and doxorubicin or addition of ifosfamide and etoposide to those who were poor responders on histopathology [46, 47]. After completion of treatment, routine follow-up evaluation was performed every 3 months for the first 2 years and every 6 months for the subsequent years. For the purpose of this study follow-up data were collected till 31st December 2020. In addition to clinical evaluation, follow-up imaging examination consisting of chest radiographs alternating with NCCT chest every 3 monthly for a total of 5 years.

### Histopathological response evaluation

Pathologist, blinded to the clinical status and MRI results, analysed resected lesions, described tumour-size, extent and amount (in percentage) of necrosis relative to the whole tumour volume. Response to NACT was assessed histologically according to the six-grade scale of Salzer-Kuntschik et al. [43]. Patients were categorised into two groups—good-response (patients with ≤ 50% viable-tumor) combining Grade I-IV and poor-response (patients with > 50% viable-tumour) combining Grade V–VI patients as Salzer-Kuntschik grading [43].

### Survival outcome evaluation

Endpoints studied were event free survival (EFS) and overall survival (OS). An event was defined as the elapse of secondary tumours or distant metastasis or local recurrence, or death from any cause. EFS was defined as the time interval from the first day of chemotherapy to any of the events or to the last date of follow-up, whichever is first. OS was defined as the time interval from the first day of chemotherapy until death. Patients who were alive without any event at the time of the last follow-up were censored and they were included both in EFS and OS.

### MRI acquisition protocol

MRI was acquired at three time-points—baseline or pre-NACT (t0), after the 1st NACT cycle (t1, 2–3 weeks) and after the 3rd NACT cycle (t2, 8–9 weeks). MRI acquisition was performed using a 1.5T Philips Achieva® MR scanner with phased-array surface coil or an extremity coil. Conventional T1-weighted, T2-weighted and IVIM-DWI sequences were acquired according to the standard MRI acquisition protocol. T1-weighted and T2-weighted images were acquired using the Turbo-Spin-Echo sequence with TR/TE = 528/10 ms and 3797/60 ms respectively, matrix-size = 512 × 512 and 384 × 384 respectively. Acquisition of IVIM-DWI was performed using free-breathing spin-echo echo-planar imaging (SE-EPI) with a variation of gradient strengths of 11 b-values (0, 10, 20, 30, 40, 50, 80, 100, 200, 400, 800 s/mm^2^) and with matrix size = 192 × 192, TR/TE = 7541/67 ms, slice-thickness/Gap = 5/0.5 mm, voxel-size = 1.3/1.3/5.0 mm, field-of-view = 250 × 250 mm^2^, and axial slices of 64. IVIM-DWI was acquired at three time-points whereas T1-weighted and T2-weighted images were acquired at time-points t0 and t2.

### Quantitative imaging parameters

Tumour-volume (in cc) at three time-points was determined using region of interest (ROI) drawn manually by an expert radiologist (D.K., > 12 years of experience in cancer imaging) covering whole tumour on b = 800 s/mm^2^ DWI images with reference to the morphological T1W and T2W images. Quantitative IVIM parameters Diffusion coefficient (*D*), Perfusion coefficient (D*) and Perfusion fraction (*f*) and *ADC* were evaluated in whole tumour-volume at three time-points t0, t1 and t2. IVIM parameters were evaluated using the state-of-the-art IVIM analysis method BE model with adaptive Total Variation (TV) Penalty function (BETV method) [39]. BETV method applies non-linear least-square (NNLS) optimisation for data fitting with adaptive penalty function Total Variation for reconstruction with good SNR and reduces the non-physiological spatial inhomogeneity in estimated parametric images. The product of relative micro-vascular flow and volume (D*.f) was also calculated voxel-wise in tumour-volume and analysed as it provides the information about vascular changes in terms of relative microvascular perfusion or blood flow, analogous to the blood-flow as measured in perfusion imaging [48]. ADC was calculated by a mono-exponential model using b-values ≥ 200 s/mm^2^ assuming perfusion effect is negligible at higher b-values [27].

Goodness-of-fit (R^2^) and Coefficient-of-variation (CV) were calculated as the measure of precision and reproducibility in IVIM parameter estimation respectively. Histogram analysis of imaging parameters was performed in tumour-volume at three time-points. Eleven (n = 11) histogram parameters: mean, standard-deviation(SD), skewness, kurtosis, energy, entropy, 90th, 75th, 60th, 50th and 25th percentiles and their relative percentage changes between time-points t0–t1(ΔI) and time-points t0–t2(ΔII) were calculated for each patient. Quantitative parameters evaluation and histogram analysis was performed using an in-house built toolbox in MATLAB® (MathWorks Inc., v2017, Philadelphia, USA).

### Clinical parameters

Tumour-volume, alkaline phosphatase (ALP), lactate dehydrogenase (LDH) at baseline, and classical prognostic factors like primary tumour site (axial/pelvic vs peripheral), presence of metastatic disease at diagnosis were evaluated for their potential impact on chemotherapy response and survival outcome as EFS and OS.

### Statistical analysis

Inter-group (between good-response and poor-response) statistical significance (*p* < 0.05) of clinical parameters and absolute histogram parameters of *ADC*, *D*, D*, f & D*.f and their relative percentage changes (ΔI & ΔII) were evaluated using independent sample t test. Intra-group significant (*p* < 0.05) changes in parameters across time-points were tested using paired t-test. Predictive performance of statistically significant (*p* < 0.05) parameters for NACT responsiveness was assessed using receiver-operating-characteristic-curve (ROC) analysis at time-points t0, and t1.

Univariate Cox regression analysis was used to assess the effects of the statistically significant (*p* < 0.05) clinical and imaging parameters on EFS and OS using hazard ratio (HR). Significant histogram parameters derived from *ADC*, *D*, D* & *f* were tested separately for multicollinearity using variance inflation factor (VIF), while VIF ≥ 8 indicated high collinearity. Using Harrells’s c-index [49] and the corresponding generalization of Somers' *D* rank correlation [50] (SDRC), the parameters with the most discriminative ability for EFS and OS was selected to develop the multivariate cox proportional hazard model in combination with significant clinical parameters. Higher values of C-index and SDRC indicated better discriminative ability. Final multivariate survival model(s) was tested for assumption of proportionality using Schoenfeld test. Kaplan–Meier curves were evaluated for the parameters showing statistical significance (*p* < 0.05) after multivariate analysis, and differences were assessed by using a log-rank test. Before inclusion in the Cox analysis, the distributions of the continuous parameters were examined for normality and a multiplicative transformation (× 10^3^$$)$$ was applied on extreme observations (mean, SD, energy, 90th–25th percentiles of imaging parameters having values in the order of 10^–3^) as reported earlier [51]. Analysis for EFS and OS was performed in all patients at time-point t0.

**Fig. 1:**
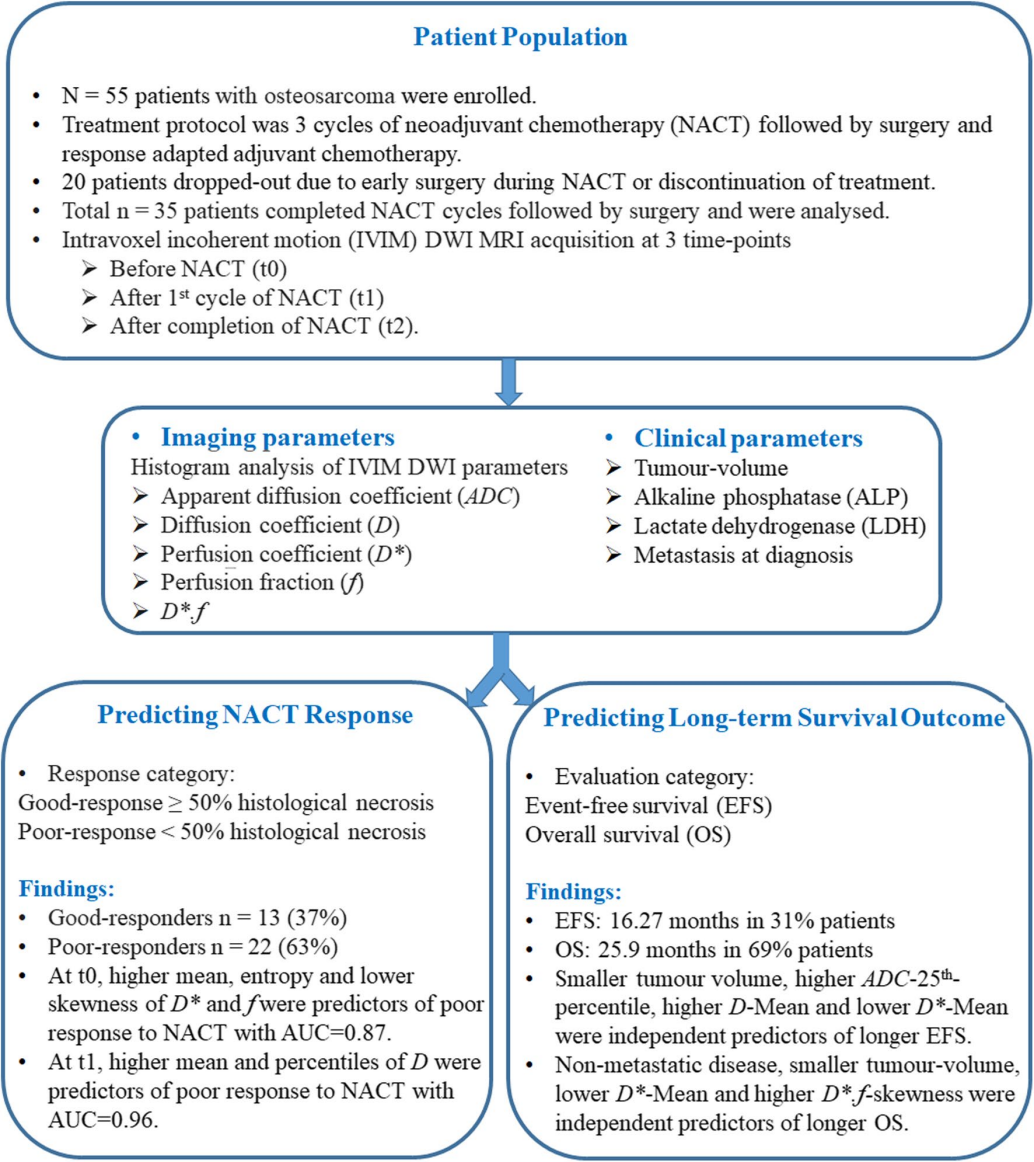
Workflow of the study

Statistical analyses were performed using SPSS v16.0 software (IBM Corporation) and R open-source statistical software (version 1.3.1073; RStudio, PBC, http://www.r-project.org). Workflow of this study is depicted in Fig. 1.

## Results

### Patient population, chemotherapy response and survival outcome

The demographic and clinical characteristics of patients at baseline are presented in Table 1. Total fifty-five (n = 55) patients were enrolled in this study. Among them 20 patients were dropped-out due to early surgery before completion of NACT cycles or discontinuation of treatment due to death or other reasons. Total thirty-five patients (n = 35; Male: Female = 27:8; Age = 18.1 ± 6.2 years; Metastatic: localized = 11:24) with osteosarcoma of conventional type were further analysed. Location of primary tumour involved femur (n = 17.64%), proximal-tibia (n = 15.43%) and humerus (n = 3.1%). After histopathological assessment, thirteen patients (n = 13.37%) were classified into good-response (GradeI: 0, GradeII: 1, GradeIII: 3, GradeIV: 9) and twenty-two patients (n = 22.63%) in poor-response (GradeV: 20, GradeVI: 2) groups. The median and range of follow-up time was 25.93 (7.6–54.4) months respectively. At the time of analysis, among 35 patients, median EFS time was 16.27 (5.5–54.4) months and median OS time was 25.9 (7.6–54.4) months and 11 (31%) patients had EFS whereas 24 patients (69%) had OS.

**Table 1:** Clinical characteristics of patients

Characteristics	Values
Age [median (range)]	16.5 (12–24) years
Sex [n (%)]	
Male	18 (75%)
Female	6 (25%)
Location of primary tumour [n (%)]	
Femur	17 (64%)
Tibia	15 (43%)
Shoulder	3 (1%)
Tumour-volume [median (range)]	412.84 (87.01–3559.46) cc
Metastasis at diagnosis [n (%)]	
Metastatic	11 (31%)
Localized	24 (69%)
Alkaline phosphatase [median (range)]	412 (134–7980) IU/L
Lactate dehydrogenase [median (range)]	303 (173–665) U/L
Histopathological response evaluation	
Percentage of necrosis [median (range)]	30 (0–99) %
Good response [n (%)]	13 (37%)
Poor response [n (%)]	22 (63%)
Survival outcome evaluation	
Follow-up time [median(range)]	25.93 (7.6–54.4) months
Event free survival [n (%), median (range) time]	11 (31%), 16.27 (5.5–54.4) months
Overall survival [n (%), median (range) time]	24 (69%), 25.9 (7.6–54.4) months

### Quantitative imaging parameters during NACT

Table 2 presents the averages of *ADC*, *D*, D*, f and D*.f and their relative percentage changes (ΔI & ΔII) in good-response (n = 13) and poor-response (n = 22) groups at three time-points. Mean R^2^ value calculated for BETV method fitting were 0.97 ± 0.03 and CV values obtained as *ADC*:29.6 ± 9.2%, *D:*30.7 ± 10.1%, D*:96.4 ± 23.2% and *f*:56.7 ± 12.9%.

**Table 2:** Average Apparent Diffusion Coefficient (*ADC*), Diffusion coefficient (*D*), Perfusion Coefficient (D*), Perfusion fraction (*f*) and D*.f in good-response (GR) and poor-response (PR) groups at baseline (t0), after 1st cycle of chemotherapy (t1), and after 3rd cycle of chemotherapy (t2) and the relative percentage changes of parameters across time-points t0 & t1 (ΔI) and t0 & t2 (ΔII)

Parameters	GR (n = 13)	PR (n = 22)	*p*-value
Time-point t0			
*ADC (*× 10^−3^ mm^2^/s)	1.30 ± 0.29*^#^	1.41 ± 0.36*^#^	0.36
*D (*× 10^−3^ mm^2^/s)	1.21 ± 0.28*^#^	1.35 ± 0.32*^#^	0.20
D* (× 10^−3^ mm^2^/s)	23.76 ± 7.56^#^	30.95 ± 10.80^#^	**0.04**
*f (%)*	13.31 ± 2.68	14.05 ± 2.99	0.47
D*.f × 10^−3^ mm^2^/s	3.85 ± 1.90^#^	5.12 ± 2.52^#^	0.13
Time-point t1			
*ADC (*× 10^−3^ mm^2^/s)	1.53 ± 0.28*	1.68 ± 0.29*	**0.04**
*D (*× 10^−3^mm^2^/s)	1.44 ± 0.35*	1.66 ± 0.27*	**0.02**
D* (× 10^−3^mm^2^/s)	19.77 ± 4.20	25.32 ± 12.47	0.13
*f (%)*	13.36 ± 2.04	12.89 ± 2.69	0.59
D*.f (× 10^−3^mm^2^/s)	2.97 ± 1.02	4.49 ± 2.32	**0.01**
*ADC-*ΔI (%)	20.08 ± 20.24	29.00 ± 31.17	0.36
*D-*ΔI (%)	21.63 ± 27.15	28.68 ± 28.12	0.47
D*-ΔI (%)	− 14.86 ± 26.63	− 10.45 ± 50.66	0.63
*f-*ΔI (%)	3.15 ± 20.28	− 5.56 ± 21.42	0.24
D*.f-ΔI (%)	− 12.12 ± 34.90	− 4.90 ± 39.22	0.59
Time-point t2			
*ADC (*× 10^−3^ mm^2^/s)	1.66 ± 0.21^#^	1.75 ± 0.30^#^	0.35
*D (*× 10^−3^ mm^2^/s)	1.57 ± 0.20^#^	1.66 ± 0.27^#^	0.29
D* (× 10^−3^ mm^2^/s)	17.77 ± 6.02^#^	23.44 ± 10.65^#^	0.09
*f (%)*	12.55 ± 1.24	13.22 ± 2.54	0.38
D*.f (× 10^−3^ mm^2^/s)	2.53 ± 1.37^#^	3.50 ± 1.86^#^	0.11
*ADC-*ΔII (%)	31.35 ± 21.99	30.01 ± 33.74	0.90
*D-*ΔII (%)	33.08 ± 18.73	27.60 ± 29.48	0.55
D*-ΔII (%)	− 22.12 ± 29.97	− 19.98 ± 31.83	0.79
*f-*ΔII (%)	− 2.16 ± 21.90	− 2.74 ± 23.86	0.94
D*.f-ΔII (%)	− 26.64 ± 49.64	− 23.73 ± 30.44	0.83

*p*-values are showing statistical significance of parameters between GR and PR groups using independent sample t test

^*^ Significantly change (*p* < 0.05) according to paired t-test between time point t0 and t1

^#^ Significantly change (*p* < 0.05) according to paired t-test between time point t0 and t2

At baseline, mean *ADC* ((1.3 vs. 1.41) × 10^−3^ mm^2^/s) and *D* ((1.21 vs. 1.35) × 10^−3^ mm^2^/s) demonstrated similar values among both the response groups with no significant difference (*p* = 0.36). Mean D* among good-responders was significantly lower than the poor-responders (D* = (23.76 ± 7.56 vs. 30.95 ± 10.80) × 10^−3^ mm^2^/s; *p* = 0.04), whereas, *f* and D*.f were not significantly different among both the response groups at baseline.

At t1, both *ADC* and *D* showed significant increase among both response groups, however, higher increase among poor-responders were observed than the good-responders (ΔI = 29%↑ vs. 20–22%↑). At t1, mean *ADC* and mean *D* values were significantly (*p* < 0.02) lower among good-responders than poor-responders. D* and D*.f were observed to decrease after 1st cycle of NACT among both response groups. At t1, mean D*.f among good-responders was significantly lower than the poor-responders (D*.f = (2.97 ± 1.02 vs. 4.49 ± 2.32) × 10^−3^ mm^2^/s; *p* = 0.01).

After t1, *ADC* and *D* did not increase any further among poor-responders, whereas in good-responders the increase in value was persistent (ΔII-ΔI = 1%↑ vs. 12%↑). D* and D*.f showed significant reduction at t2 in comparison to baseline values. However, in the course of NACT, comparatively a higher reduction in D*(ΔI = 15% vs. 10%; ΔII = 22% vs. 20%) and D*.f (ΔI = 12% vs. 5%; ΔII = 27% vs. 24%) were observed among good-responders than the poor-responders. During NACT, *f* did not change significantly among both the response groups; however, *f-mean* showed reduction among good-responders as well as poor-responders after NACT.

### Histogram Analysis of IVIM parameters during NACT

Detailed comparison of histogram parameters between good-response and poor-response groups is presented in Additional file 1: Table S1. At baseline, D*-skewness (1.22 vs. 0.6; *p* = 0.04) was significantly higher and D*-entropy (8.27 vs 9.2; *p* = 0.04) and D*-90th-percentile ((60.83 ± 18.23 vs. 72.14 ± 8.1) × 10^−3^ mm^2^/s; *p* = 0.03) were significantly lower among good-responders than poor-responders. At baseline, *f-entropy* was significantly lower among good-responders (8.5 vs. 9.1; *p* = 0.04) and significantly lower D*.f-entropy (6.79 vs. 7.63; *p* = 0.03) were observed among good-responders than poor-responders.

At t1, 90th percentile of *ADC* and 90th–25th percentile values of *D* were significantly (*p* < 0.02) lower among good-responders than poor-responders. Histograms of *ADC* and *D* became more negatively skewed among good-responders than poor-responders (*ADC-skewness*: − 0.37 vs. − 0.18; *D-skewness*: − 0.31 vs. − 0.29) after NACT. During NACT, D* and D*.f histograms were more peaked and positively skewed among good-responders than that of poor-responders (D*-skewness:1.5 vs. 1.2; D*.f-skewness: 3.32 vs. 3.1).

**Fig. 2:**
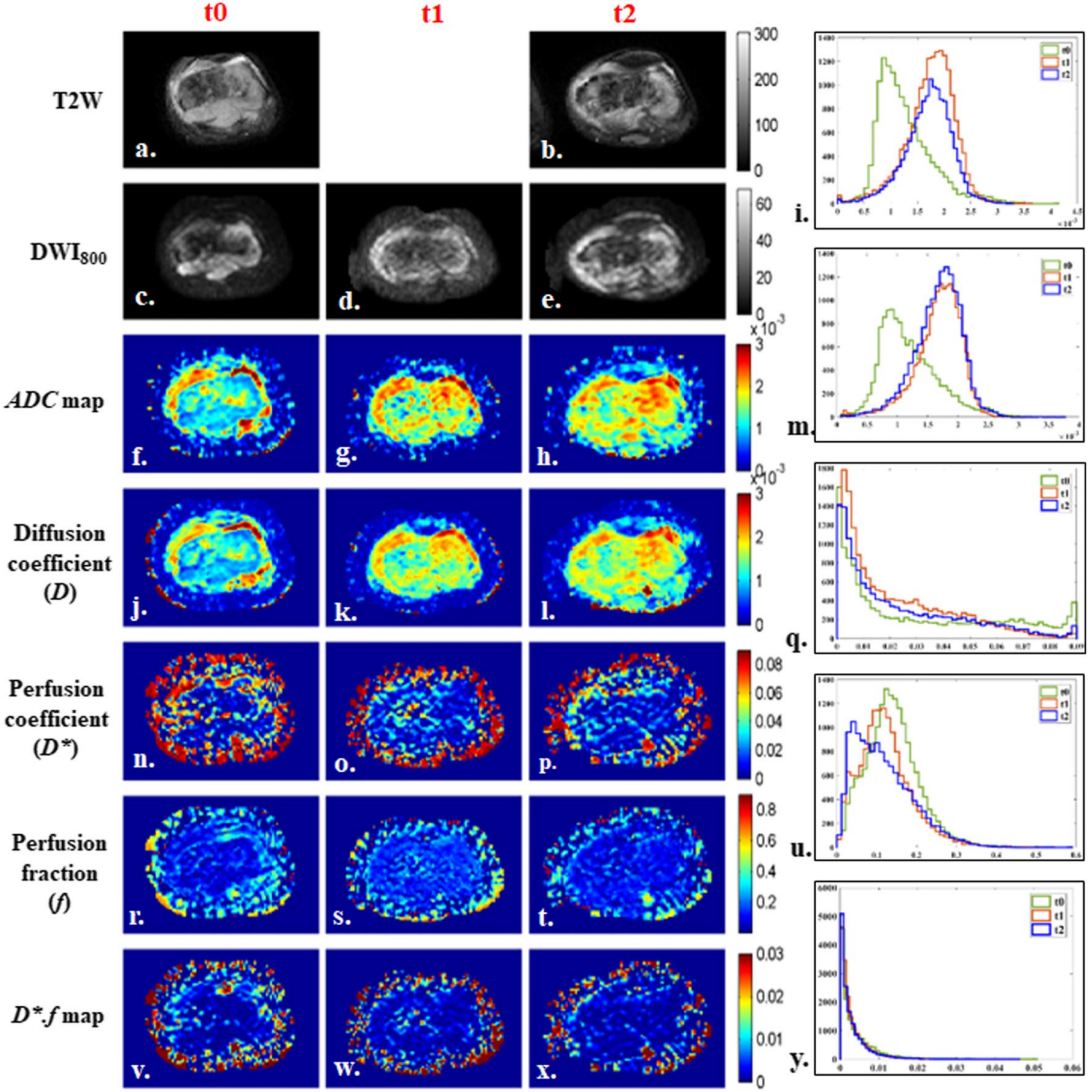
18 years old male patient from good-response group, with localized osteosarcoma of 284 cc volume in right distal femur at baseline. Patient had > 90% histological necrosis after surgery and event free survival and overall survival of 54 months. 1st, 2nd and 3rd columns show images at time points t0 (baseline), t1 (after 1st cycle of chemotherapy) and t2 (after completion of chemotherapy) respectively and 4th column represents histograms of parametric maps in tumor volume at three time-points t0 (green), t1 (orange) and t2 (blue). **a**, **b** T2-weighted fat saturated image, **c**–**e** DWI (b = 800 s/mm^2^), **f**–**h** Apparent diffusion coefficient (*ADC*), **i** Histogram of *ADC* was high peaked and sharp at t0, moved towards the right of the coordinate and became wider at t1 and t2. **j**–**l** Diffusion coefficient (*D*), **m** Histogram of *D* was high peaked and sharp at t0, moved towards the right of the coordinate at t1 and t2. **n**–**p** Perfusion coefficient (D*), **q** Histogram of D* was highly peaked and positively skewed with a heavy tail at t0, became more positively skewed with a lighter tail at t1 and t2. **r**–**t** Perfusion fraction (*f*), **u** Histogram of *f* was highly peaked at t0 and became more positively skewed and wider at t1 and t2. **v**–**x** D*.f, **y** Histogram of D*.f was high peaked at t0 and became more positively skewed and high peaked at t1 and t2

**Fig. 3:**
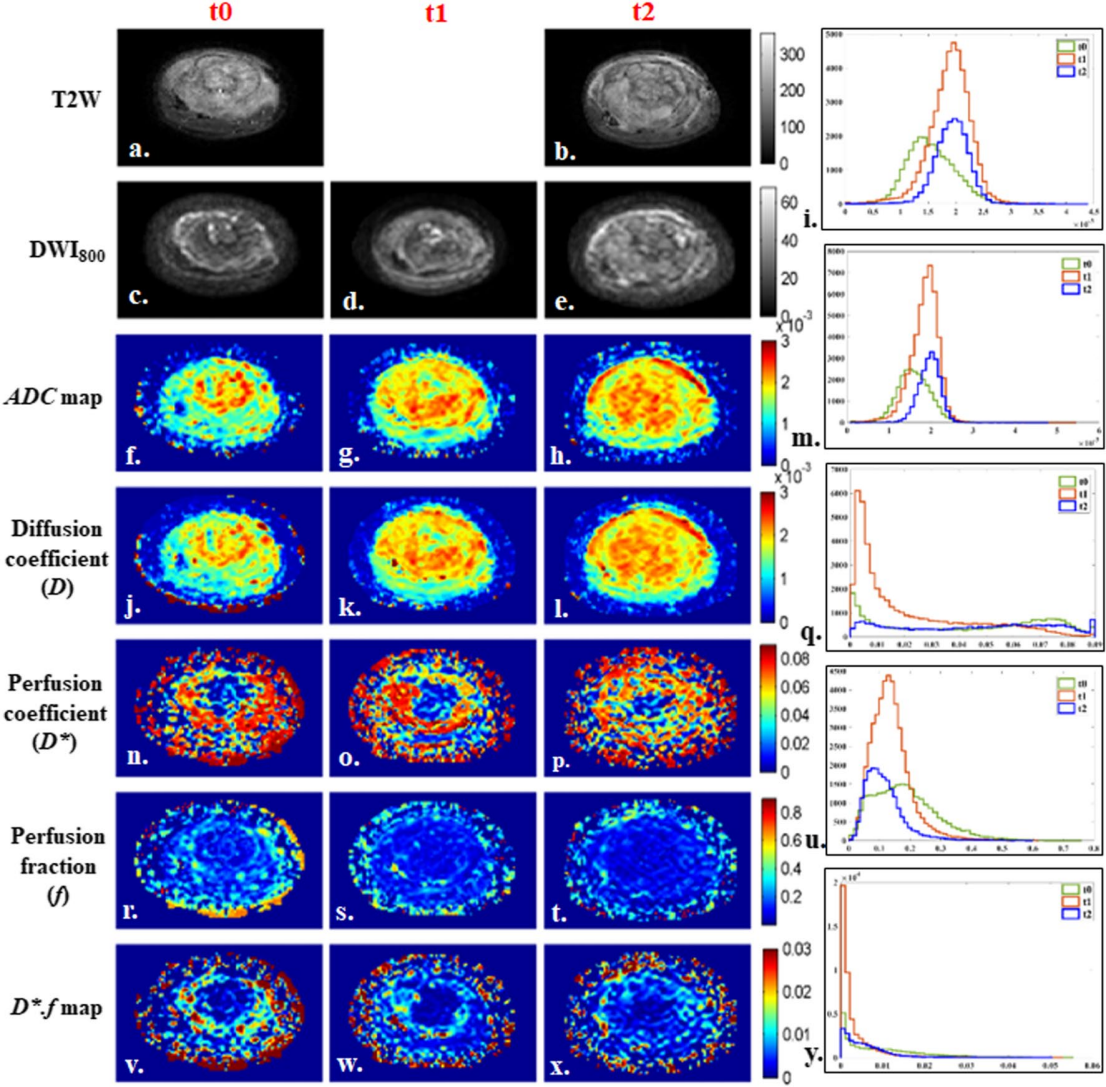
12 years old male patient from poor-response group, with osteosarcoma of 554 cc volume in left tibia and multiple metastatic lung nodules at baseline. Patient had 35% histological necrosis in resected tumor specimen and event free survival and overall survival of 16.3 months. 1st, 2nd and 3rd columns show images at time points t0 (baseline), t1 (after 1st cycle of chemotherapy) and t2 (after completion of chemotherapy) respectively and 4th column represents histograms of parametric maps in tumor volume at three time-points t0 (green), t1 (orange) and t2 (blue). **a**, **b** T2-weighted fat saturated image, **c**–**e** DWI (b = 800 s/mm^2^), **f**–**h** Apparent diffusion coefficient (*ADC*), **i** Histogram of *ADC* was high peaked and sharp at t0 and slightly shifted to the right of the coordinate and became sharply peaked at t1 and t2. **j**–**l** Diffusion coefficient (*D*), **m** Histogram of *D* was high peaked and sharp at t0 and slightly shifted to the right of the coordinate and became sharply peaked at t1 and t2. **n**–**p** Perfusion coefficient (D*), **q** Histogram of D* was positively skewed with a heavy tail at t0, became highly peaked at t1 and turned to a wider & flat (low peaked) shape at t2. **r**–**t** Perfusion fraction (*f*), **u** Histogram of *f* was wide and low peaked at t0, became slightly positively skewed and high peaked at t1 and t2. **v**–**x** D*.f, **y** Histogram of D*.f was low peaked with long tail at t0 and became high peaked at t1 and more positively skewed at t2

**Fig. 4:**
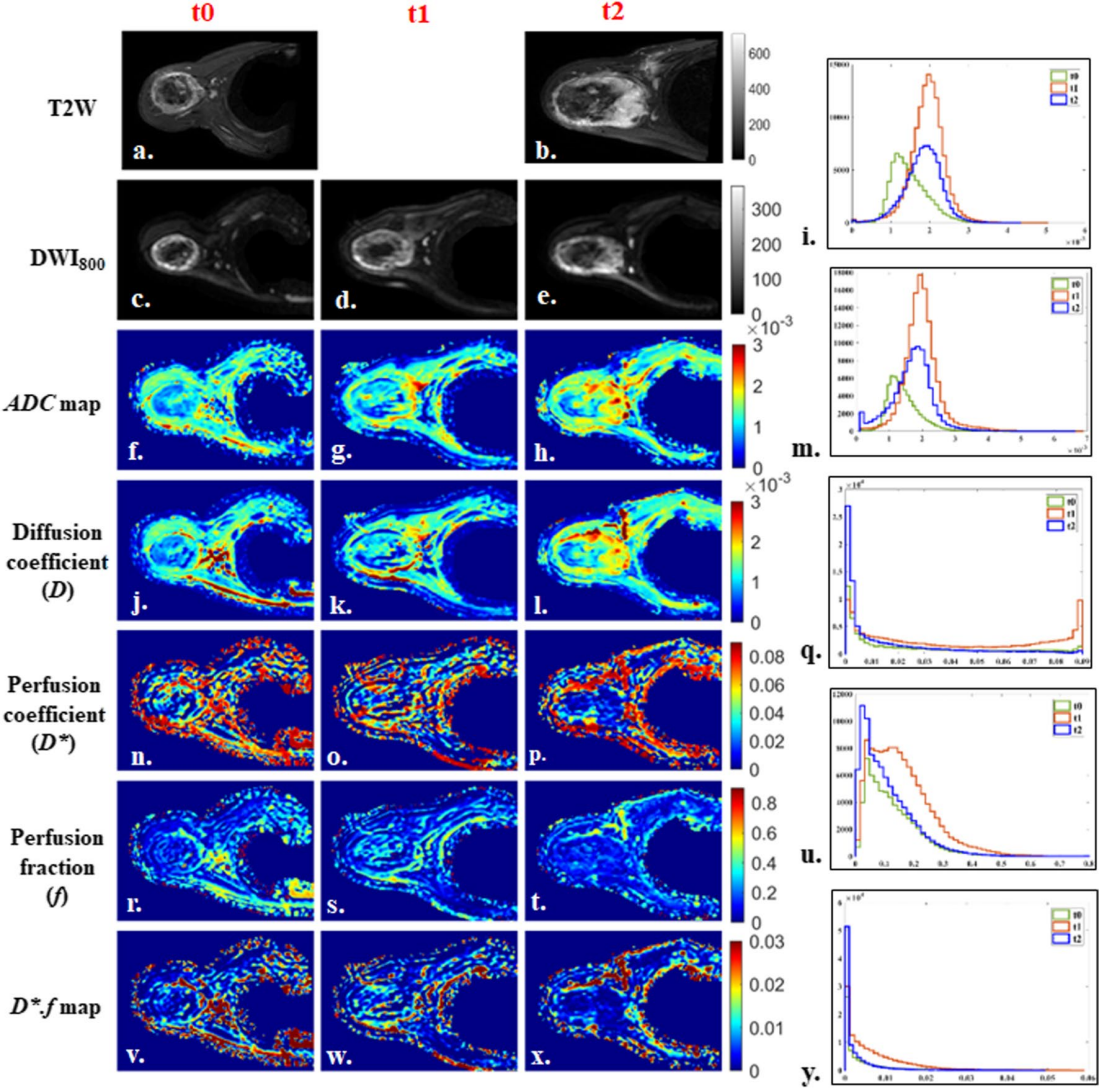
14 years old male patient from good-response group, with localized osteosarcoma of 330 cc volume in right proximal humerus at baseline. Patient had > 50% histological necrosis after surgery and event free survival and overall survival of 22.5 months. 1st, 2nd and 3rd columns show images at time points t0 (baseline), t1 (after 1st cycle of chemotherapy) and t2 (after completion of chemotherapy) respectively and 4th column represents histograms of parametric maps in tumor volume at three time-points t0 (green), t1 (orange) and t2 (blue). **a**, **b** T2-weighted fat saturated image, **c**–**e** DWI (b = 800 s/mm^2^), **f**–**h** Apparent diffusion coefficient (*ADC*), **i** Histogram of *ADC* was high peaked and sharp at t0, moved towards the right of the coordinate and became wider at t1 and t2. **j**–**l** Diffusion coefficient (*D*), **m** Histogram of *D* was high peaked and sharp at t0, moved towards the right of the coordinate at t1 and t2. n, **o**, **p** Perfusion coefficient (D*), **q** Histogram of D* was positively skewed at t0, became wider & flat (low peaked) shape at t1, and turned to high peaked with a long tail at t2. **r**–**t** Perfusion fraction (*f*), **u** Histogram of *f* was wide and low peaked at t0, became slightly positively skewed and high peaked at t1 and t2. **v**–**x** D*.f, **y** Histogram of D*.f was low peaked with long tail at t0 and t1 and became high peaked and more positively skewed at t2

Illustrative example of IVIM parametric maps and corresponding histograms in tumor volume of representative patients with tumor involving different anatomical regions like femur, tibia and humerus are presented in Figs. 2, 3 and 4 respectively. No significant qualitative or quantitative reginal differences was observed in the IVIM parametric maps evaluated in different anatomical regions.”

### Chemotherapy response prediction

**Fig. 5:**
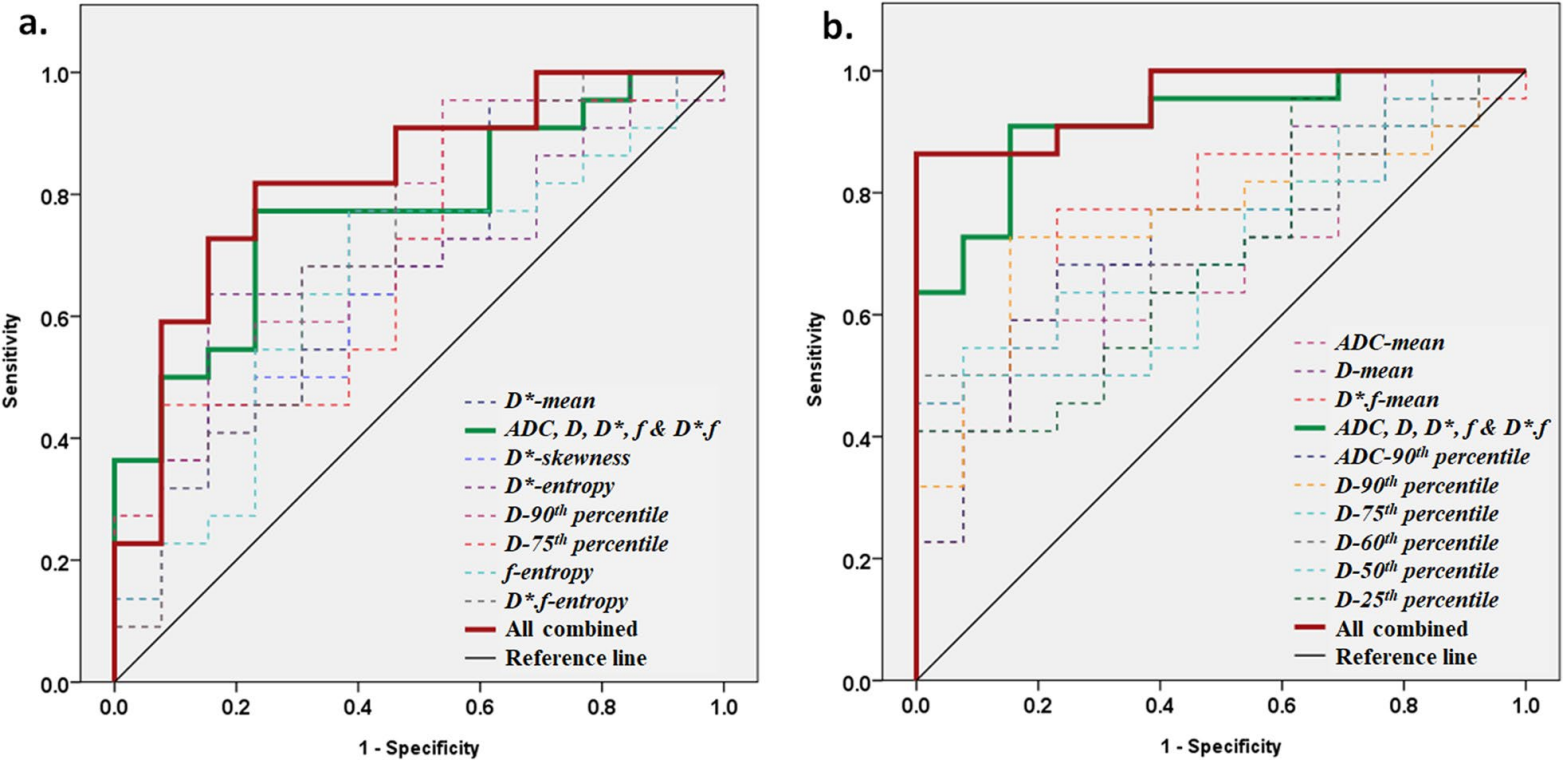
ROC curve analysis using mean and statistically significant (*p* < 0.05) histogram parameters of ADC, D, D*, *f* and D*.f **a** before commencement of chemotherapy (time-point t0) and **b** after 1st cycle of neoadjuvant chemotherapy (time-point t1). Mean parameters jointly showed AUC = 0.77, sensitivity = 77%, specificity = 77% at t0 and AUC = 0.92, sensitivity = 91%, specificity = 85% at t1; however, in combination with histogram parameters produced AUC = 0.87, sensitivity = 86%, specificity = 77% at t0 and AUC = 0.96, sensitivity = 86%, specificity = 100% at t1 in predicting poor-response to chemotherapy

Clinical parameters like tumour-volume, ALP, LDH, primary tumour site and metastasis were not found to be statistically significant (*p* > 0.13) between good-response and poor-response groups. Statistically significant imaging parameters and their ROC curve analysis for chemotherapy response prediction is presented in Table 3. Mean of imaging parameters individually showed AUCs of 0.57–0.7, and in combination showed AUC = 0.77 in predicting poor-response at t0. Statistically significant (*p* < 0.05) histogram parameters D*-skewness, D*-entropy, D*-90th- percentile, f-entropy and D*.f-entropy jointly with mean of *ADC*,*D*,D*,f &D*.*f* showed AUC = 0.87 with Sensitivity = 86% and Specificity = 77% in predicting poor-response at t0 (Fig. 5a). At t1, mean of imaging parameters *ADC,D*,D*,f &D*.f jointly showed AUC = 0.92 in predicting poor-response; while in combination with statistically significant (*p* < 0.05) histogram parameters *ADC-90th-percentile* and *D-90th–25th-percentiles* produced AUC = 0.96 with Sensitivity = 86% and Specificity = 100%; in predicting poor-response to NACT (Fig. 5b).

**Table 3:** Statistically significant (independent sample t test, *p* < 0.05) histogram parameters of ADC, D, D*, f and D*.f among good-response and poor-response groups at baseline (time-points t0) and after 1st cycle of chemotherapy (time-point t1) and their ROC curve analysis for predicting poor-response to chemotherapy

Time-point	Parameters	t test, *p* value	Sensitivity (%)	Specificity (%)	Cut-off Value	AUC (95% CI)
t0	*ADC-Mean*	0.36	55	67	> 1.36	0.61 (0.42–0.80)
	*D-Mean*	0.20	65	67	> 1.2	0.66 (0.47–0.85)
	D*-Mean	0.04	65	75	> 26.9	0.70 (0.52–0.87)
	*f-Mean*	0.47	75	50	> 12.04	0.57 (0.37–0.77)
	D*.f-Mean	0.13	65	67	> 3.95	0.67 (0.48–0.86)
	Mean ADC, D, D*, f, D*.f combined		77	77	< 0.58	0.77 (0.62–0.93)
	D*-skewness	0.04	71	67	< 0.27	0.7 (0.52–0.88)
	D*-entropy	0.04	68	69	> 8.72	0.7 (0.52–0.88)
	D*-90th percentile	0.03	77	62	> 64.68	0.72 (0.54–0.9)
	D*-75th percentile	0.04	76	57	> 36.05	0.7 (0.52–0.88)
	*f-entropy*	0.04	77	62	> 8.77	0.65 (0.46–0.85)
	D*.f-entropy	0.03	68	69	> 7.1	0.72 (0.53–0.9)
	All Combined		**86**	**77**	**< 0.66**	**0.87 (0.68–0.99)**
t1	*ADC-mean*	0.04	60	77	> 1.72	0.71 (0.57–0.9)
	*D-mean*	0.02	68	70	> 1.62	0.73 (0.54–0.89)
	D*-mean	0.13	64	77	> 21.33	0.65 (0.46–0.83)
	*f-mean*	0.59	60	58	> 12.32	0.61 (0.42–0.80)
	D*.f-mean	0.01	77	77	> 3.14	0.76 (0.59–0.92)
	Mean ADC, D, D*, f, D*.f combined		**91**	**85**	**> 0.55**	**0.92 (0.82–1)**
	*ADC-90th percentile*	0.03	68	**77**	> 2.26	0.72 (0.54–0.89)
	*D-90th percentile*	0.01	73	85	> 2.17	0.76 (0.59–0.92)
	*D-75th percentile*	0.03	64	**77**	> 1.94	0.72 (0.56–0.89)
	*D-60th percentile*	0.04	64	62	> 1.77	0.70 (0.53–0.88)
	*D-50th percentile*	0.04	55	62	> 1.68	0.70 (0.53–0.88)
	*D-25th percentile*	0.03	55	69	> 1.36	0.71 (0.53–0.89)
	All Combined		**86**	**100**	**> 0.60**	**0.96 (0.89–1)**

SD: Standard deviation; ROC: Receiver Operating Characteristics Curve; AUC: Area under the Curve; CI: confidence interval

*ADC*: × 10^−3^ mm^2^/s *D*: × 10^−3^ mm^2^/s; D*: × 10^−3  ^mm^2^/s*; f:* %. D*.f: × 10^−3^mm^2^/s

### Survival outcome prediction

Baseline parameters from univariate and multivariate Cox regression analyses which had significant effects on EFS and OS are presented in Table 4. Univariate analyses showed, clinical parameters such as metastasis, tumour-volume and ALP were found to be significantly associated with EFS and OS.

**Table 4:** Clinical and imaging parameters from univariate and multivariate Cox regression analysis that have statistically significant (*p* < 0.05) association with the event free survival (EFS) and overall survival (OS) in patients with osteosarcoma (n = 35)

EFS (n = 35)			OS (n = 35)		
Parameters	HR (95% CI)	***p*** value	Parameters	HR (95% CI)	***p*** value
*Univariate analysis*					
Clinical parameter			Clinical parameter		
Metastasis	2.9 (1.2–6.6)	0.013	Metastasis	5.4 (2.1–14)	< 10^–3^
ALP	1 (0.99–1.001)	0.005	ALP	1 (0.99–1.002)	0.002
Tumour-volume	1 (0.99–1.01)	0.011	Volume	1 (0.99–1.002)	0.013
Imaging parameter			Imaging parameter		
*ADC*-Mean	0.14 (0.028–0.74)	0.02	D*-Mean	1.1 (1–1.1)	0.009
*ADC*-90th percentile	0.27 (0.075–0.96)	0.043	D*-skewness	0.41 (0.2–0.83)	0.014
*ADC*-75th percentile	0.22 (0.06–0.84)	0.026	D*-90th percentile	1.1 (1–1.1)	0.032
*ADC*-60th percentile	0.21 (0.051–0.86)	0.03	D*-75th percentile	1 (1–1.1)	0.017
*ADC*-50th percentile	0.19 (0.042–0.87)	0.033	D*-60th percentile	1 (1–1.1)	0.013
*ADC*-25th percentile	0.11 (0.017–0.7)	0.019	D*-50th percentile	1 (1–1.1)	0.010
*D*-Mean	0.12 (0.019–0.79)	0.027	D*-25th percentile	1.1 (1–1.1)	0.023
*D*-90th percentile	0.17 (0.035–0.84)	0.03	D*.f-skewness	0.56 (0.34–0.94)	0.028
*D*-75th percentile	0.19 (0.04–0.88)	0.034	D*.f-kurtosis	0.93 (0.87–1)	0.045
*D*-60th percentile	0.19 (0.038–0.96)	0.044	D*.f-entropy	1.8 (1–3)	0.033
*D*-50th percentile	0.18 (0.034–0.99)	0.049	D*.f-60th percentile	1.2 (1–1.3)	0.042
*D*-25th percentile	0.18 (0.041–0.82)	0.026	D*.f-50th percentile	1.2 (1–1.4)	0.030
D*-Mean	1.1 (1–1.1)	0.004	D*.f-25th percentile	1.6 (1.1–2.3)	0.021
D*-skewness	0.37 (0.18–0.76)	0.007	–		
D*-75th percentile	1 (1–1.1)	0.013	–		
D*-60th percentile	1 (1–1.1)	0.007	–		
D*-50th percentile	1 (1–1.1)	0.004	–		
D*-25th percentile	1.1 (1–1.2)	0.001	–		
D*.f-60th percentile	1.2 (1–1.3)	0.032	–		
D*.f-50th percentile	1.2 (1–1.5)	0.015	–		
D*.f-25th percentile	2 (1.3–3.1)	0.002	–		
*Multivariate analysis*					
EFS-Model-1			OS-Model-1		
Tumour-volume	1.002 (1.001–1.003)	0.001	Metastasis	4.330 (1.638–11.449)	< 10^–3^
* ADC*-25th percentile	0.047 (0.006–0.394)	0.005	D*-Mean	1.045 (0.999–1.093)	0.056
EFS-Model-2			OS-Model-2		
Tumour-volume	1.001 (1–1.003)	0.007	Metastasis	2.955 (1.021–8.554)	0.046
*D*-Mean	0.1 (0.014–0.726)	0.023	Tumour-volume	1.001 (1–1.003)	0.042
D*-Mean	1.052 (1.003–1.103)	0.039	D*.f-skewness	0.544 (0.298–0.995)	0.048

**Fig. 6:**
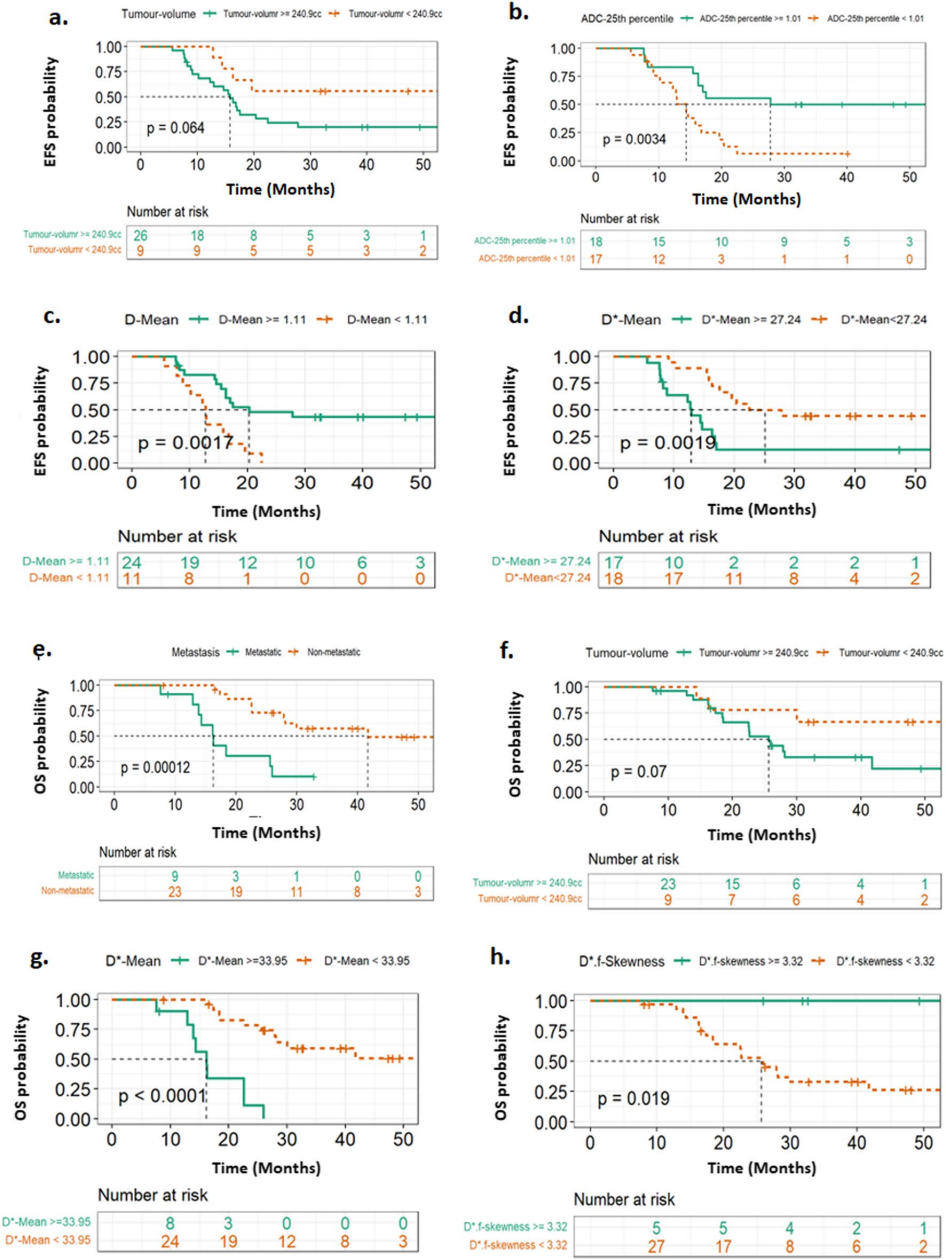
Kaplan–Meier survival curves demonstrate differences in patient outcome groups at a cut-off value by log-rank test for event free survival (EFS) and overall survival (OS). **a** Tumor volume for EFS; difference was *p* = 0.064 at a cut-off value of 240.9 cc tumor volume. **b** *ADC*-25th percentile^#^ for EFS; difference was *p* = 0.0034 at a cut-off value of 1.01. **c** *D*-Mean^#^ for EFS; difference was *p* = 0.0017 at a cut-off value of 1.11. **d** D*-Mean^#^ for EFS; difference was *p* = 0.0019 at a cut-off value of 27.24. **e** Metastasis for OS; significant difference was *p* < 10^–3^ by log-rank test. **f** Tumor volume for OS; the significant difference was *p* = 0.07 at a cut-off value of 240.9 cc. **g** D*-Mean^#^ for OS; difference was *p* < 10^–3^ at a cut-off value of 33.95. **h** D*.f-skewness^#^ for OS; difference was *p* = 0.019 at a cut-off of 3.32. ^#^ Parameter values were transformed by multiplying with 10^3^ before analysis

Imaging parameters *ADC*-Mean, *ADC*-90th–25th percentiles, *D*-Mean, *D*-90th–25th percentiles, D*-Mean, D*-skewness, D*-75th–25th percentiles and D*.f-60th–25th percentiles were significantly (*p* < 0.05) associated with EFS. These parameters derived from *ADC*, *D* and D* respectively had high VIF scores (≥ 220, ≥ 70, ≥ 32 respectively) and after comparing the c-index and SDRC values (details are in Additional file 1: Table S2), *ADC*-25th percentile, *D*-mean and D*-Mean were selected to develop multivariate model in combination with significant clinical parameters. As *ADC*-25th percentile and *D*-Mean had high VIF scores (> 8), both the parameters were tested separately along with the other parameters (Metastasis, ALP, tumour-volume, D*-Mean) as two separate models for multivariate cox analysis. Hazard ratio forest plots for multivariate analysis are depicted in Additional file 1: Fig. S1a, b respectively. Two cox-proportional hazard model were developed such as, EFS-Model-1 including tumour-volume (HR = 1.002, *p* = 0.001) and *ADC*-25th percentile (HR = 0.047, *p* = 0.005) and EFS-Model-2 including tumour-volume (HR = 1.001, *p* = 0.007), *D*-Mean (HR = 0.1, *p* = 0.023) and D*-Mean (HR = 1.052, p = 0.039) parameters that showed significant and independent association with EFS. Both EFS-Model-1 and EFS-Model-2 met the requirement of proportionality of the covariates (global Schoenfeld test *p*-value = 0.57, 0.19 respectively, Additional file 1: Fig. S2a, b), however, EFS-Model-2 was observed to had comparatively higher discriminative power than EFS-Model-1 (c-index = 0.728 vs. 0.686 and standard-error = 0.061 vs. 069). Kaplan–Meier curve for tumour-volume, *ADC*-25th percentile, *D*-Mean and D*-mean against EFS probability is shown in Fig. 6a–d respectively. Log-rank test results showed probability of EFS was lower in patients with larger tumour-volume (cut-off = 240.9 cc, *p* = 0.064), lower level of *ADC*-25th percentile (cut-off = 1.01, *p* = 0.003), *D*-Mean (cut-off = 1.11, *p* = 0.002) and higher D*-Mean (cut-off = 27.24, *p* = 0.002) at baseline.

Univariate analysis for OS showed, imaging parameters D*-Mean, D*-skewness, D*-90th–25th percentiles, D*.f-skewness, D*.f-kurtosis, D*.f-entropy, D*.f-60th–25th percentiles were significant (*p* < 0.05). These parameters were derived from D* and thus had high VIF scores and after comparing the c-index and SDRC values (Details are in Additional file 1: Table S3), D*-mean, D*-skewness, D*-75th percentile and D*.f-skewness parameters were selected and tested separately in combination with significant clinical parameters (Metastasis, ALP, tumour-volume) to develop multivariate cox proportional hazard models. Hazard ratio forest plots for multivariate analysis are depicted in Additional file 1: Fig. S3a, b respectively. Two cox-proportional hazard model were developed such as, OS-Model-1 including metastasis (HR = 5.409, *p* < 10^–3^) and D*-Mean (HR = 1.045, *p* = 0.056) and OS-Model-2 including metastasis (HR = 2.995, *p* = 0.046), tumour-volume (HR = 1.001, *p* = 0.042) and D*.f-skewness (HR = 0.544, *p* = 0.048) parameters that showed significant and independent association with OS. Both OS-Model-1 and OS-Model-2 met the requirement of proportionality of the covariates (global Schoenfeld test p-value = 0.31, 0.53 respectively, Additional file 1: Fig. S6a, b). Both the models OS-Model-1 and OS-Model-2 produced comparable discriminative power for OS (c-index = 0.743 vs. 0.736 and standard-error = 0.061 vs. 064). Kaplan–Meier curves for metastasis, tumour-volume, D*-mean and D*.f-skewness against OS probability are depicted in Fig. 6e–h respectively. Log-rank test results showed OS probability was significantly lower in patients with metastatic disease (*p* < 10^–3^), larger tumour-volume (cut-off = 240.9 cc, *p* = 0.07) and higher levels of D*-mean (cut-off = 33.95, *p* < 10^–3^) and lower D*.f-skewness (cut-off = 3.32, *p* = 0.019) at presentation.

## Discussion

This prospective study evaluates the role of non-invasive quantitative IVIM imaging in characterizing tumour microenvironment, predicting chemotherapy response and long-term survival outcome of osteosarcoma at baseline and early in the course of treatment.

Histogram analysis of IVIM-DWI parameters showed, microvascular perfusion and its heterogeneity in tumour were significantly higher among poor-responders than good-responders characterizing higher angiogenic changes among poor-responders, thus effectively predicted poor-response to NACT at baseline (t0, AUC = 0.87). While after the first cycle of NACT, diffusion parameters along with D*.f predicted poor-response to NACT with high AUC (t1, AUC = 0.96).

For survival outcome, multivariate Cox regression analysis showed smaller tumour volume, higher levels of *ADC*-25th percentile, *D*-Mean and lower D*-Mean as the independent predictor of EFS; while nonmetastatic disease, smaller tumour volume, lower levels of D*-Mean and higher D*.f-skewness were independent predictors of OS. These findings suggest quantitative IVIM parameters and their histogram analyses may be useful for characterizing and quantifying heterogeneity in tumour micro-environment and thereby predicting chemotherapeutic response and outcome in osteosarcoma.

IVIM imaging is influenced by the diffusion of free water molecules in the intra & extra-cellular compartments and micro-circulation of water molecules in micro-capillaries. The observations in this study support that cell density and abnormal/immature micro-vessels may decrease in osteosarcoma after chemotherapy resulting an increase in *ADC* and *D* and reduction in D* and *f* similar to the previous studies in osteosarcoma [11–15, 37, 38] and other tumours like colorectal [30], head and neck [31], cervical [32], nasopharyngeal [33, 34], breast [35], hepatic [36] and other tumours [28, 29].

At baseline, similar mean *ADC* and *D* values were observed among both the response groups possibly due to high heterogeneity of cellularity in osteosarcoma, similar to the previous studies [11, 14, 15, 37]. After 1st NACT cycle, mean *ADC* and *D* values in tumour were significantly higher among poor-responders than the good-responders; however, after an initial increase in *ADC* and *D* at t1 (ΔI≈29%↑), diffusion did not increase any further (ΔII≈ΔI≈29%↑) among poor-responders. Necrotic tumours with large extracellular space (with higher mean and percentile values of *ADC*&*D*) are often associated with poor response to therapy [12, 31, 34] due to hypoxia and tissue acidosis that leads to resistance to chemotherapy [4, 52]. Whereas, for good-responders, *ADC* and *D* both were observed to be increased throughout all NACT cycles (ΔI = 20%↑, 22%↑ respectively and ΔII = 31%↑, 33%↑ respectively) indicating possible increase in cell death resulting in an increase in diffusion of water molecules in the tumour. At baseline, higher levels of diffusion parameters were observed to be associated with improved EFS in osteosarcoma patients similar to earlier studies in other tumours [28, 29, 36].

IVIM perfusion related parameters (D*, f, D*.f) correlate with the process of angiogenesis and reflect changes in relative microvascular perfusion, perfusion volume-fraction and flow in tumour respectively [48]. Lee et al. showed that, D* and *f* were significantly correlated with the micro-vessel density score in murine model colorectal cancer; providing information about tumour perfusion and angiogenesis [53]. In this study, a higher heterogeneity in micro-perfusion pattern in osteosarcoma was observed as the markers of therapeutic poor response similar to previous studies [18, 19, 25, 26, 44]. On the other hand, comparatively a higher reduction in D* and D*.f during chemotherapy and lower heterogeneity in tumour microvasculature among good-responders indicated relatively lower angiogenic progression. Lower levels of perfusion parameters were associated with improved EFS and OS in osteosarcoma patients similar to earlier studies in other tumours [28, 29, 36]. Analysis showed, perfusion parameters (D* & *f)* had higher predictive values (AUC = 0.6–0.8) than diffusion parameters (*ADC & D)* (AUC = 0.6–0.7) at baseline and after the 1st cycle of chemotherapy in predicting NACT response.

*ADC* value has been widely used for assessing chemotherapy response and survival outcome in osteosarcoma [11–17]. However, in this study, IVIM parameters in combination with *ADC* showed improved prediction performance for chemotherapy response than *ADC* alone (t0, AUC = 0.77 vs. 0.61; t1, AUC = 0.92 vs. 0.71). Measurement of true tissue diffusion was observed to be necessary and useful for characterizing chemotherapeutic changes in osteosarcoma, similar to other IVIM studies in literature [27, 28, 30, 31]. The ability of IVIM to characterise early changes in microvascular perfusion along with true diffusion in tumour is highly relevant in this era of anti-angiogenic chemotherapy drugs which can be performed without the use of exogenous contrast agent; however, reliability and reproducibility should be ensured. Widely used BE model [27, 32] and segmented-BE techniques [30, 31, 33, 35] evaluates IVIM parameters at each voxel independently, overlooking the spatial context that may lead to unreliable solutions resulting in noisy parametric image reconstruction, especially for perfusion related parameters [29, 54]. Thus, adding a physiologically plausible spatial constraint to the existing BE model is expected to provide a reliable parametric estimation as shown by Baidya Kayal et al. [39]. This recently developed BETV method [39] incorporates gradient based penalty TV [55] with NNLS optimisation of the BE model to preserve the desired spatial homogeneity in the parametric images. Robustness of this method has been shown earlier in both cancer simulations & clinical cohorts of osteosarcoma [39], Ewing sarcoma [40], lymphoma [41], brain tumour [42] and prostate [43]. In this study, the-state-of-the-art BETV method was effectively used for analysing IVIM-DWI acquired with a 1.5T scanner and able to provide potential imaging biomarkers for NACT response and survival outcome in osteosarcoma with satisfactory results.

There are a few limitations of our study. First, among the available pathological scales to categorise chemotherapeutic response groups, in this study, patients were categorised into good-response (≥ 50% HPE-necrosis) and poor-response(< 50% HPE-necrosis) groups with reference to the earlier study by Picci et al. [56]. Secondly, perfusion or functional imaging, as DCE MRI or FDG-PET/CT, would have been beneficial to characterise angiogenesis changes after treatment and validate the findings from IVIM perfusion parameters; however, we could not do contrast MRI due to financial and time constraints considering the fact that our study protocol involved three time-point imaging for each patient. Thirdly, advanced texture analysis might be helpful, but that would expand the scope of current study considerably. Texture analysis and its usefulness can be dealt separately in future studies. Fourthly, different IVIM analysis methods like Bayesian based or stretched exponential methods that may hold relevance for clinical assessment of osteosarcoma; however, these methods were not evaluated in the current study. Finally, as osteosarcoma is a rare tumour, only a limited number of patients (35 patients) could be analysed in this prospective study. Thus, future studies analysing much larger cohort and multi-centric data with standardized MRI protocol could be useful.

## Conclusions

In conclusion, clinical parameters such as tumour volume, nonmetastatic disease and ALP can be independent predictors of survival outcome. IVIM diffusion (*D*) and perfusion-related parameters (D*, f) and their histogram analysis (skewness, entropy, percentile) indicating heterogeneity in micro-vasculature in tumour are useful imaging markers to predict survival outcome at presentation and non-response to NACT before and early in the course of treatment. Therefore, quantitative IVIM analysis with advanced analysis methods can serve as a surrogate marker for characterizing chemotherapeutic response, which can be used for non-invasive monitoring and evaluation of chemotherapy response and treatment outcome in patients with osteosarcoma.

## Supplementary Information

**Additional file 1. **Additional Tables and figures.

## Data Availability

The datasets generated and analysed during the current study are not publicly available due data privacy policy of the institute but are available from the corresponding author on reasonable request.

## Abbreviations

*ADC*: Apparent diffusion coefficient
ALP: Alkaline phosphatase
AUC: Area under the curve
BETV: Bi-exponential model with Total Variation Penalty function
*D*: Diffusion coefficient
*D**: Perfusion coefficient
DWI: Diffusion weighted imaging
EFS: Event free survival
*f*: Perfusion fraction
HPE: Histopathological evaluation
HR: Hazard ratio
IVIM: Intravoxel incoherent motion
LDH: Lactate dehydrogenase
NACT: Neoadjuvant chemotherapy
OS: Overall survival
ROC: Receiver operating characteristics
SD: Standard deviation
t0: Baseline
t1: After 1-cycle of neoadjuvant chemotherapy
t2: After 3-cycles of neoadjuvant chemotherapy
TV: Total variation
ΔI: Relative percentage change from t0 to t1
ΔII: Relative percentage change from t0 to t2
