# Supplementary material for: Non-invasive intravoxel incoherent motion MRI in prediction of histopathological response to neoadjuvant chemotherapy and survival outcome in osteosarcoma at the time of diagnosis
Source: J Transl Med. 2022 Dec 27;20:625. doi: 10.1186/s12967-022-03838-1 (PMC9795762; doi:10.1186/s12967-022-03838-1)

# Additional file

| **Table S1:** Histogram parameters of Apparent diffusion coefficient (*ADC)*, Diffusion-coefficient *(D)*, Perfusion-coefficient *(D*)* *&* Perfusion-fraction *(f)* observed in different response groups: Good-response (GR) and poor-response (PR) at time points t0 (before chemotherapy), t1 (after 1st cycle of chemotherapy) and t2 (after completion of chemotherapy) and their relative percentage changes between time points t0 & t1 (ΔI) and t0 & t2 (ΔII). Significant values ($p$<0.05) are in bold. | | | | | | | | | | |
|---|---|---|---|---|---|---|---|---|---|---|
| | **Good-response** (n=13) | | | | | **Poor-response** (n=22) | | | | |
| | **t0** | **t1** | **t2** | **ΔI (%)** | **ΔII (%)** | **t0** | **t1** | **t2** | **ΔI (%)** | **ΔII (%)** |
| ***ADC*** | | | | | | | | | | |
| Mean | **1.30±0.29*#** | **1.53±0.28*†** | **1.66±0.21#** | 20.08±20.24 | 31.35±21.99 | **1.41±0.36*#** | **1.74±0.32*†** | **1.75±0.30#** | 29.0±31.17 | 30.01±33.74 |
| SD ($10^{-1}$) | 4.1±0.06 | 4.37±0.06 | 4.7±0.04 | 7.87±17.94 | 21.09±34.06 | 4.6±1.1 | 4.41±1.03 | 4.55±1.26 | -1.18±19.72 | 3.35±27.52 |
| Skewness | **0.87±0.51*#** | **0.09±0.56*** | **-0.27±0.43#** | -72.74±72.42 | -112.3±85.95 | **0.57±0.67*#** | **-0.12±0.74*** | **-0.22±0.60#** | -31.98±54.66 | -56.83±284.8 |
| Kurtosis | 4.50±1.57 | 3.86±1.70 | 3.38±0.63 | -8.61±39.17 | -15.82±34.14 | 4.41±2.15 | 4.21±1.24 | 4.01±0.98 | 6.80±41.29 | 1.58±36.17 |
| Energy ($10^{-3}$) | 1.45±0.33 | 1.22±0.44 | 1.14±0.25 | -14.20±28.51 | -17.79±26.74 | 1.59±1.12 | 1.26±0.33 | 1.49±0.78 | -6.22±28.68 | 17.25±86.04 |
| Entropy | 6.75±0.21 | 6.92±0.31 | 6.96±0.23 | 2.34±4.16 | 2.99±4.35 | 6.86±0.30 | 6.89±0.25 | 6.83±0.30 | 0.59±3.17 | -0.28±5.14 |
| 90th percentile | **1.85±0.29*#** | **2.09±0.29*†** | **2.25±0.25#** | 13.84±12.71 | 24.06±22.05 | **2.02±0.43*#** | **2.29±0.32*†** | **2.32±0.30#** | 17.37±22.31 | 18.61±23.85 |
| 75th percentile | **1.53±0.32*#** | **1.82±0.30*** | **2.0±0.22#** | 21.41±19.03 | 34.96±24.76 | **1.68±0.42*#** | **2.03±0.35*** | **2.06±0.33#** | 26.58±33.75 | 29.06±35.63 |
| 60th percentile | **1.33±0.32*#** | **1.64±0.30*** | **1.81±0.21#** | 25.74±23.33 | 40.66±25.29 | **1.47±0.41*#** | **1.85±0.36*** | **1.86±0.33#** | 32.76±38.60 | 34.40±41.31 |
| 50th percentile | **1.23±0.32*#** | **1.53±0.30*** | **1.69±0.21#** | 27.69±25.83 | 42.44±25.58 | **1.36±0.39*#** | **1.74±0.36*** | **1.75±0.34#** | 35.38±39.57 | 36.85±43.16 |
| 25th percentile | **1.02±0.30*#** | **1.24±0.29*** | **1.33±0.26#** | 26.39±30.32 | 36.61±27.78 | **1.10±0.33*#** | **1.68±0.29*** | **1.46±0.35#** | 39.81±39.54 | 41.62±46.13 |
| ***D*** | | | | | | | | | | |
| Mean | **1.21±0.28*#** | **1.44±0.28*†** | **1.57±0.20#** | 21.63±27.15 | 33.08±18.73 | **1.35±0.32*#** | **1.68±0.29*†** | **1.66±0.27#** | 28.68±28.12 | 27.60±29.48 |
| SD ($10^{-1}$) | 4.5±1.1 | 4.65±1.0 | 4.75±1.05 | 6.56±17.91 | 11.76±36.99 | 4.5±1.2 | 4.32±1.1 | 4.3±1.1 | -0.89±20.32 | 0.44±28.20 |
| Skewness | **0.62±0.48*#** | **0.03±0.64*** | **-0.23±0.51#** | -155.8±163.35 | -207.8±293.9 | **0.54±0.74*#** | **-0.12±0.76*** | **-0.31±0.51#** | -282.67±142.9 | -179.4±299.5 |
| Kurtosis | 4.23±1.50 | 3.97±1.10 | 3.82±0.88 | 1.23±33.61 | -1.81±34.60 | 4.81±2.48 | 4.72±1.96 | 4.22±1.54 | 10.71±49.92 | 0.08±40.74 |
| Energy ($10^{-3}$) | 1.77±3.5 | 1.45±0.59 | 1.31±0.35 | 0.02±57.67 | -11.24±37.64 | 2.24±2.96 | 1.45±0.48 | 1.54±0.63 | -8.49±37.96 | -3.0±47.75 |
| Entropy | 6.64±0.51 | 6.86±0.19 | 6.83±0.25 | 4.01±10.68 | 3.67±11.06 | 6.75±0.27 | 6.79±0.34 | 6.73±0.36 | 0.58±4.45 | -0.19±5.58 |
| 90th percentile | **1.80±0.22*#** | **2.01±0.23*†** | **2.13±0.19#** | 12.31±12.48 | 20.08±17.51 | **1.93±0.36*#** | **2.21±0.28*†** | **2.19±0.24#** | 16.59±18.50 | 15.79±18.62 |
| 75th percentile | **1.47±0.28*#** | **1.74±0.27*†** | **1.89±0.18#** | 19.95±18.97 | 31.57±19.76 | **1.62±0.37*#** | **1.96±0.31*†** | **1.96±0.27#** | 24.89±28.43 | 25.01±28.28 |
| 60th percentile | **1.27±0.29*#** | **1.56±0.30*†** | **1.72±0.19#** | 24.99±24.22 | 38.88±20.91 | **1.42±0.36*#** | **1.79±0.33*†** | **1.78±0.3#** | 31.46±33.60 | 31.14±35.19 |
| 50th percentile | **1.16±0.30*#** | **1.45±0.31*†** | **1.60±0.21#** | 27.41±27.76 | 42.04±21.63 | **1.32±0.35*#** | **1.69±0.33*†** | **1.68±0.31#** | 34.43±35.34 | 34.02±38.21 |
| 25th percentile | **0.89±0.38*#** | **1.13±0.34*†** | **1.27±0.26#** | 104.38±305.1 | 93.08±183.54 | **1.05±0.32*#** | **1.42±0.32*†** | **1.40±0.32#** | 41.94±40.99 | 41.68±46.89 |
| ***D**** | | | | | | | | | | |
| Mean | **23.76±7.56#†** | 19.77±4.20 | **17.76±6.02#** | -14.86±26.63 | -18.93±32.52 | **30.95±10.80#†** | 25.32±12.47 | **23.44±10.65#** | -10.45±50.65 | -20.05±31.78 |
| SD | 23.69±4.26 | 21.93±3.09 | 20.46±5.12 | -5.41±18.57 | -9.84±33.56 | 26.01±3.24 | 22.38±6.18 | 22.22±5.96 | -12.27±27.46 | -13.40±23.41 |
| Skewness | **1.10±0.60†** | 1.42±0.51 | 1.65±0.68 | 73.99±133.01 | 132.6±253.8 | **0.67±0.65†** | 1.23±1.07 | 1.20±0.70 | 44.18±208.63 | 227.9±334.8 |

| | | | | | | | | | | |
|---|---|---|---|---|---|---|---|---|---|---|
| Kurtosis | 3.58±1.79 | 4.48±2.43 | 5.76±3.50 | 38.03±51.79 | 100.8±145.6 | 2.61±1.30 | 5.21±5.41 | 4.19±2.54 | 129.01±260.22 | 72.93±102.28 |
| Energy ($10^{-3}$) | 0.41±0.32 | 0.71±1.09 | 0.65±0.51 | 108.52±385.9 | 186.2±233.5 | 1.15±3.51 | 0.34±0.37 | 0.44±0.51 | 130.21±140.1 | 225.2±238.9 |
| Entropy | **8.46±0.89**$^{\dagger}$ | 8.67±0.67 | 8.12±0.80 | 3.75±16.59 | -3.51±9.71 | **9.08±0.78**$^{\dagger}$ | 8.86±0.91 | 8.58±0.91 | -2.0±11.32 | -5.01±11.69 |
| 90$^{th}$ percentile | **60.28±14.15**$^{\#\dagger}$ | 55.43±10.33 | **49.59±16.35**$^{\#}$ | -3.40±29.10 | -11.58±41.65 | **70.64±11.25***$^{\#\dagger}$ | **58.28±21.58*** | **57.91±18.58**$^{\#}$ | -14.92±35.63 | -16.42±26.76 |
| 75$^{th}$ percentile | **38.71±15.69**$^{\#\dagger}$ | 29.72±9.16 | **26.26±13.31**$^{\#}$ | -15.19±34.12 | -22.43±45.68 | **49.85±16.69***$^{\#\dagger}$ | **39.18±21.74*** | **36.05±17.79**$^{\#}$ | -11.84±60.03 | -22.83±34.95 |
| 60$^{th}$ percentile | **23.14±11.82**$^{\#}$ | 15.86±6.91 | **14.16±8.41**$^{\#}$ | -16.42±44.01 | -27.30±43.79 | **33.56±18.27***$^{\#}$ | **26.14±18.33*** | **22.43±15.41**$^{\#}$ | 7.08±117.93 | -18.99±60.46 |
| 50$^{th}$ percentile | 14.92±9.14 | 10.29±5.61 | 8.84±5.27 | -10.11±57.92 | -23.05±49.48 | 24.56±17.04 | 19.33±15.42 | 15.96±13.65 | 31.19±94.92 | -10.66±73.38 |
| 25$^{th}$ percentile | 4.28±3.74 | 3.53±2.26 | 2.97±1.77 | -8.39±41.27 | -12.49±78.23 | 8.35±8.54 | 6.97±6.84 | 5.75±5.95 | 95.94±179.54 | 26.62±164.04 |
| ***f*** | | | | | | | | | | |
| Mean | 13.31±2.68 | 13.36±2.04 | 12.55±1.24 | 3.15±20.28 | -2.16±21.90 | 14.05±2.99 | 12.89±2.69 | 13.22±2.54 | -5.56±21.42 | -2.74±23.86 |
| SD | 8.25±2.63 | 8.12±3.30 | 7.68±1.73 | -0.16±25.84 | -2.62±45.07 | **8.53±3.21*** | **6.81±1.98*** | 7.12±1.74 | -14.22±26.75 | -6.61±33.21 |
| Skewness | 0.95±0.33 | 1.03±0.30 | 1.07±0.42 | 15.49±40.23 | 23.49±53.03 | 1.08±0.41 | 0.96±0.43 | 1.08±0.47 | 21.08±132.94 | 72.87±119.23 |
| Kurtosis | 4.82±2.07 | 5.19±1.55 | 5.52±2.59 | 14.13±29.17 | 21.92±46.09 | 5.34±1.67 | 5.40±2.12 | 5.86±2.05 | 9.14±55.91 | 18.66±54.38 |
| Energy ($10^{-3}$) | 0.19±0.10 | 0.26±0.30 | 0.30±0.20 | 50.48±219.6 | 83.85±156.6 | 0.48±0.95 | 0.21±0.23 | 0.29±0.23 | 29.51±137.5 | 133.6±160.6 |
| Entropy | **8.66±0.90**$^{\dagger}$ | 9.07±0.77 | 8.53±0.74 | 6.13±19.05 | -0.96±8.74 | **9.06±0.69**$^{\dagger}$ | 8.99±0.81 | 8.64±0.79 | -0.54±7.27 | -4.33±8.77 |
| 90$^{th}$ percentile | 24.17±5.86 | 23.69±6.22 | 22.02±4.37 | -0.86±17.02 | -5.25±27.98 | 25.06±6.45 | 21.53±5.15 | 22.33±4.55 | -10.24±23.92 | -5.81±27.49 |
| 75$^{th}$ percentile | 18.24±3.95 | 18.00±3.93 | 16.54±2.54 | 0.08±16.37 | -6.26±22.61 | 18.68±4.26 | 16.81±3.94 | 17.21±3.42 | -7.11±22.73 | -4.01±25.48 |
| 60$^{th}$ percentile | 14.48±2.94 | 14.32±2.04 | 13.44±1.45 | 1.59±19.34 | -3.82±21.75 | 14.92±3.45 | 13.92±3.14 | 14.17±2.97 | -3.79±22.73 | -1.52±25.72 |
| 50$^{th}$ percentile | 12.23±2.67 | 12.24±1.37 | 11.70±0.92 | 4.90±28.05 | 0.63±26.91 | 12.82±3.08 | 12.24±2.75 | 12.43±2.76 | -1.56±22.85 | 0.39±26.42 |
| 25$^{th}$ percentile | 7.07±2.13 | 7.12±1.84 | 7.03±1.02 | 6.31±35.30 | 8.21±35.73 | 7.77±2.32 | 7.83±2.4 | 7.89±2.30 | 5.93±26.07 | 8.60±33.03 |
| ***D*.f*** | | | | | | | | | | |
| | **t0** | **t1** | **t2** | **ΔI (%)** | **ΔII (%)** | **t0** | **t1** | **t2** | **ΔI (%)** | **ΔII (%)** |
| Mean | **3.85±1.90**$^{\#}$ | **2.97±1.02**$^{\dagger}$ | **2.53±1.37**$^{\#}$ | -12.12±34.90 | -26.64±49.64 | **5.12±2.52***$^{\#}$ | **4.49±2.31**$^{\dagger}$***** | **3.50±1.86**$^{\#}$ | -4.90±39.22 | -23.72±30.43 |
| SD | 4.97±2.15 | 4.37±1.81 | 4.32±1.73 | -6.30±35.25 | 3.72±73.41 | 5.81±1.76 | 4.30±1.80 | 4.36±1.47 | -21.66±32.99 | -19.48±28.79 |
| Skewness | 2.80±1.11 | 3.24±0.84 | 3.40±0.64 | 25.69±38.05 | 37.82±58.30 | 2.27±0.93 | 2.91±1.44 | 3.02±0.83 | 47.80±105.12 | 53.84±77.26 |
| Kurtosis | 15.86±10.10 | 19.33±8.32 | 19.68±6.84 | 52.05±82.71 | 78.81±145.24 | 11.33±7.16 | 18.68±14.77 | 17.46±7.33 | 123.9±252.6 | 105.75±141.12 |
| Energy ($10^{-3}$) | 3.82±3.73 | 4.97±7.40 | 4.32±2.63 | 74.54±214.8 | 155.5±240.8 | 3.08±4.57 | 2.68±2.85 | 2.56±2.11 | 161.8±379.8 | 167.7±308.8 |
| Entropy | **6.88±0.85**$^{\#\dagger}$ | 6.88±0.42 | **6.54±0.62**$^{\#}$ | 1.47±15.12 | -4.02±11.26 | **7.55±0.78**$^{\#\dagger}$ | 7.16±0.87 | **6.96±0.63**$^{\#}$ | -4.31±14.53 | -7.12±10.70 |
| 90$^{th}$ percentile | 10.02±5.69 | 8.34±3.88 | 7.46±3.57 | -3.50±42.69 | -2.60±75.03 | **12.55±5.09***$^{\#}$ | **9.49±4.73*** | **8.31±3.95**$^{\#}$ | -16.58±44.47 | -25.03±39.12 |
| 75$^{th}$ percentile | 5.29±3.21 | 4.67±2.42 | 3.37±1.81 | -8.80±42.62 | -16.43±60.41 | **7.21±4.02***$^{\#}$ | **5.3±3.74*** | **4.50±2.96**$^{\#}$ | -8.90±59.83 | -25.64±41.71 |
| 60$^{th}$ percentile | **2.92±1.85**$^{\#}$ | 1.97±0.60 | **1.71±0.91**$^{\#}$ | -8.55±47.75 | -22.54±45.61 | **4.50±3.23**$^{\#}$ | 3.43±3.02 | **2.68±2.31**$^{\#}$ | 3.58±88.45 | -24.29±43.92 |
| 50$^{th}$ percentile | 1.87±1.33 | 1.24±0.49 | 1.05±0.52 | -0.67±68.67 | -19.29±51.46 | **3.17±2.63**$^{\#}$ | 2.49±2.50 | **1.90±1.91**$^{\#}$ | 15.9±118.5 | -19.27±53.82 |
| 25$^{th}$ percentile | 0.50±0.50 | 0.41±0.29 | **0.30±0.22**$^{\dagger}$ | -0.56±75.27 | -16.38±85.26 | 0.98±1.06 | 0.84±1.11 | **0.69±0.81**$^{\dagger}$ | 26.74±133.16 | 54.38±136.22 |

Mean, SD (Standard-Deviation), 90$^{th}$, 70$^{th}$, 50$^{th}$ and 25$^{th}$ percentiles values of *ADC, D, D* and D*.f* are in x$10^{-3}$mm$^2$/s; Mean, SD (Standard-Deviation), 90$^{th}$, 70$^{th}$, 50$^{th}$ and 25$^{th}$ percentiles values of *f* are in percentage (%).

* Intra-group significant change ($p$<0.05) according to paired t-test between time point t0 and t1

$^{\#}$ Intra-group significant change ($p$<0.05) according to paired t-test between time point t0 and t2

$^{\dagger}$ Inter-group significant difference ($p$<0.05) according to independent sample t test at individual time points t0, t1 and t2

| **Table S2:** Variance inflation factor (VIF), Harrell's c-index and the corresponding generalization of Somers' *D* rank correlation (SDRC) for statistically significant ($p<0.05$) imaging parameters by univariate cox regression analyses for association with Event free survival (EFS) in patient cohort. | | | | |
|---|---|---|---|---|
| **Parameters** | **VIF score** | **c-index** | **SDRC** | **p-value** |
| **Derived from Apparent diffusion coefficient (ADC)** | | | | |
| ADC-Mean | 1587 | 0.640 | 0.279 | 0.046 |
| ADC-90$^{th}$ percentile | 220 | 0.630 | 0.260 | 0.050 |
| ADC-75$^{th}$ percentile | 1613 | 0.626 | 0.252 | 0.081 |
| ADC-60$^{th}$ percentile | 8336 | 0.632 | 0.264 | 0.061 |
| ADC-50$^{th}$ percentile | 4825 | 0.62 | 0.241 | 0.087 |
| ADC-25$^{th}$ percentile | 759 | 0.644 | 0.287 | 0.049 |
| **Derived from Diffusion coefficient (D)** | | | | |
| D-Mean | 336 | 0.642 | 0.283 | 0.049 |
| D-90$^{th}$ percentile | 95 | 0.640 | 0.279 | 0.040 |
| D-75$^{th}$ percentile | 1172 | 0.620 | 0.241 | 0.099 |
| D-60$^{th}$ percentile | 4822 | 0.636 | 0.272 | 0.069 |
| D-50$^{th}$ percentile | 2442 | 0.632 | 0.264 | 0.081 |
| D-25$^{th}$ percentile | 70 | 0.624 | 0.249 | 0.085 |
| **Derived from Perfusion coefficient (D*)** | | | | |
| D*-Mean | 396 | 0.322 | -0.356 | 0.001 |
| D*-skewness | 35 | 0.678 | 0.356 | 0.001 |
| D*-75$^{th}$ percentile | 380 | 0.322 | -0.356 | 0.001 |
| D*-60$^{th}$ percentile | 715 | 0.339 | -0.322 | 0.004 |
| D*-50$^{th}$ percentile | 821 | 0.335 | -0.329 | 0.003 |
| D*-25$^{th}$ percentile | 44 | 0.337 | -0.326 | 0.003 |
| D*.f-60$^{th}$ percentile | 297 | 0.356 | -0.287 | 0.014 |
| D*.f-50$^{th}$ percentile | 467 | 0.366 | -0.268 | 0.017 |
| D*.f-25$^{th}$ percentile | 32 | 0.331 | -0.337 | 0.003 |

| **Table S3:** Variance inflation factor (VIF), Harrell's c-index and the corresponding generalization of Somers' *D* rank correlation (SDRC) for statistically significant ($p<0.05$) imaging parameters by univariate cox regression analyses for association with Overall survival (OS) in patient cohort. | | | | |
|---|---|---|---|---|
| **Parameters** | **VIF score** | **c-index** | **SDRC** | **p-value** |
| **Derived from Apparent diffusion coefficient (ADC)** | | | | |
| **---** | | | | |
| **Derived from Diffusion coefficient (D)** | | | | |
| **---** | | | | |
| **Derived from Perfusion coefficient (D*)** | | | | |
| D*-Mean | 5563 | 0.308 | -0.385 | $<10^{-3}$ |
| D*-skewness | 80 | 0.676 | 0.351 | 0.002 |
| D*-90$^{th}$ percentile | 266 | 0.312 | -0.375 | 0.004 |
| D*-75$^{th}$ percentile | 600 | 0.308 | -0.385 | 0.001 |
| D*-60$^{th}$ percentile | 1437 | 0.356 | -0.288 | 0.012 |
| D*-50$^{th}$ percentile | 1247 | 0.349 | -0.303 | 0.010 |
| D*-25$^{th}$ percentile | 262 | 0.341 | -0.317 | 0.006 |
| D*.f-skewness | 103 | 0.630 | 0.259 | 0.060 |
| D*.f-kurtosis | 59 | 0.627 | 0.254 | 0.055 |
| D*.f-entropy | 5 | 0.349 | -0.303 | 0.016 |
| D*.f-60$^{th}$ percentile | 372 | 0.368 | -0.264 | 0.025 |
| D*.f-50$^{th}$ percentile | 598 | 0.361 | -0.278 | 0.014 |
| D*.f-25$^{th}$ percentile | 37 | 0.339 | -0.322 | 0.003 |

**Fig. S1:** Hazard ratio Forest plot for multivariate cox regression analysis in patient cohort (n=35) for Event free survival (EFS) for a) EFS-Model-1 and b) EFS-Model-2.

**a. Hazard ratio for event free survival analysis for EFS-Model-1**

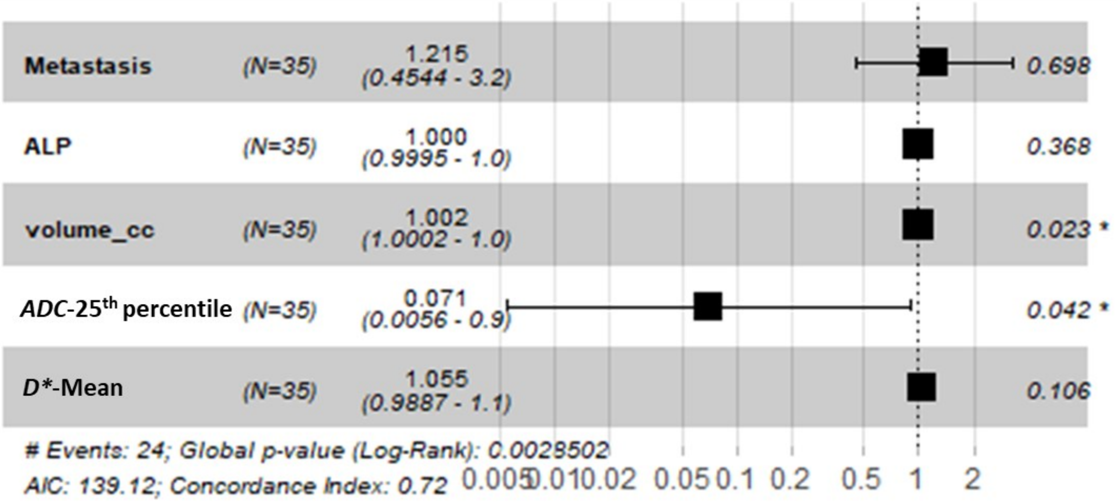


**b. Hazard ratio for event free survival analysis for EFS-Model-2**

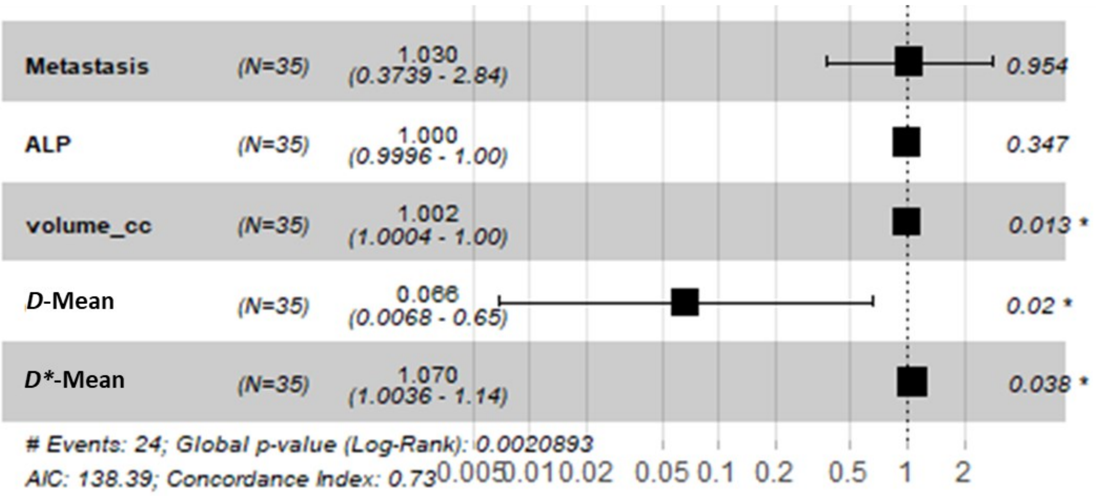

**Fig. S2:** Schoenfeld residual test to check proportional hazard function for Event free survival (EFS) for a) EFS-Model-1 (Tumour-volume, *ADC*-25th percentile) and b) EFS-Model-2 (Tumour-volume, *D*-Mean, *D**-Mean). This test infers that the hazards run proportionally to each other when the *p*-values are large ($p$>0.05). The proportionality assumption is met for both the models as all covariates are between the residuals and the parallel lines. (volume_cc: Tumour-volume; adc12: *ADC*-25th percentile; d1: *D*-Mean; dp1: *D**-Mean).

**a. EFS-Model-1**

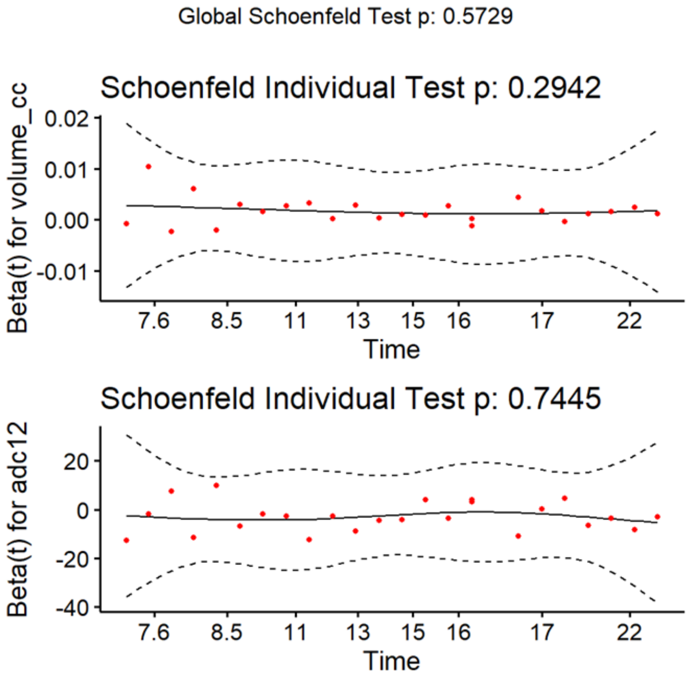


**b. EFS-Model-2**

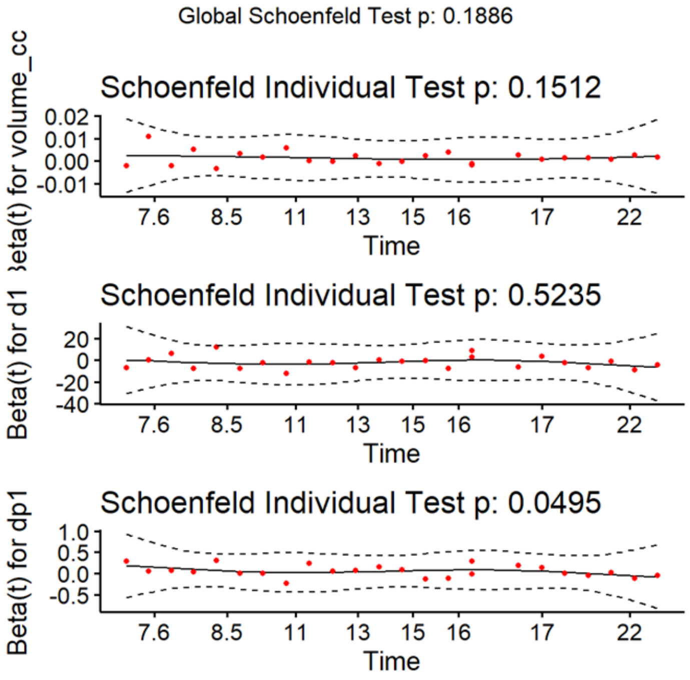

**Fig. S3:** Hazard ratio Forest plot for multivariate cox regression analysis in patient cohort (n=35) for overall survival (OS) for a) OS-Model-1 and b) OS-Model-2.

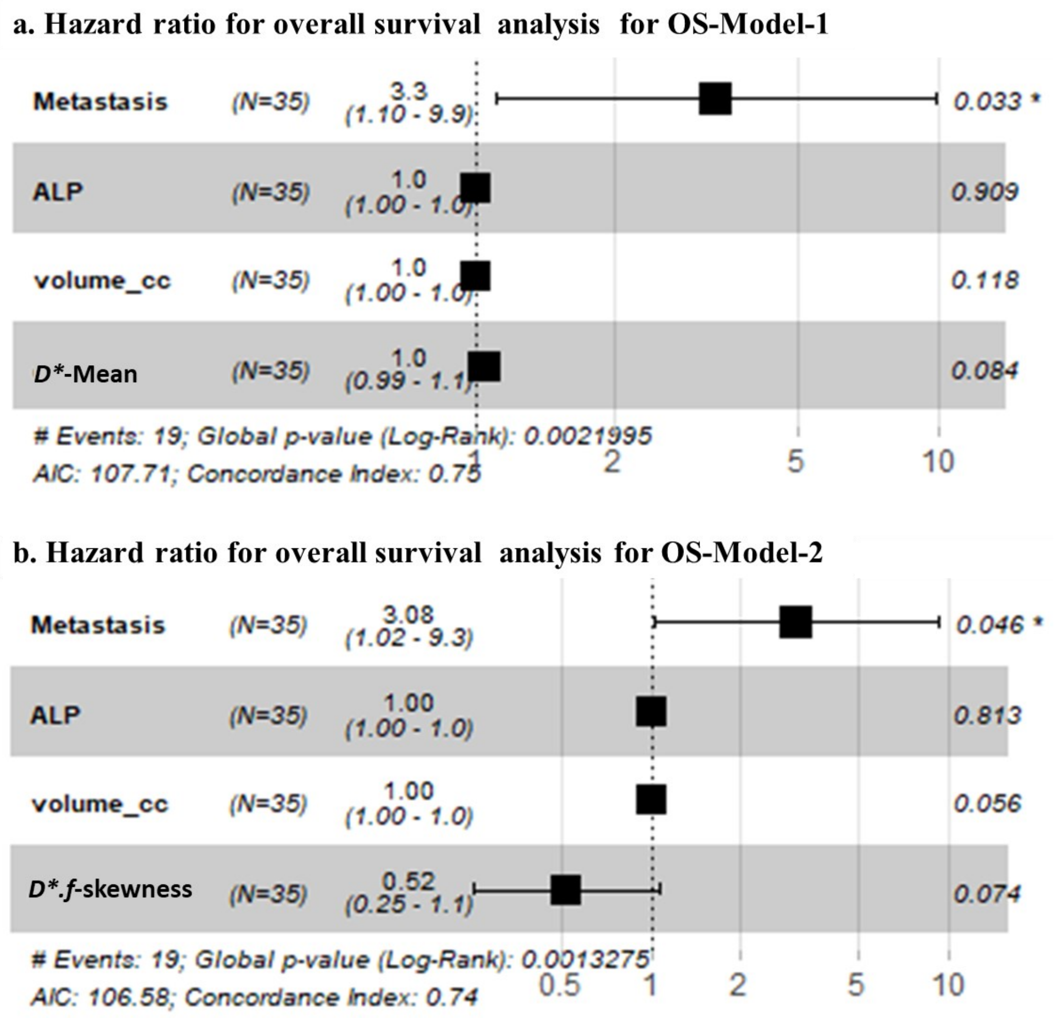

**Fig. S4:** Schoenfeld residual test to check proportional hazard function for Overall survival (OS) for a) OS-Model-1 (Metastasis, *D**-Mean) and b) OS-Model-2 (Metastasis, Tumour-volume, *D**.*f*-Skewness). This test infers that the hazards run proportionally to each other when the *p*-values are large ($p$>0.05). The proportionality assumption is met for both the models as all covariates are between the residuals and the parallel lines. (volume_cc: Tumour-volume; dp1: *D**-Mean; dpf4: *D**.*f*-skewness).

**a. OS-Model-1**

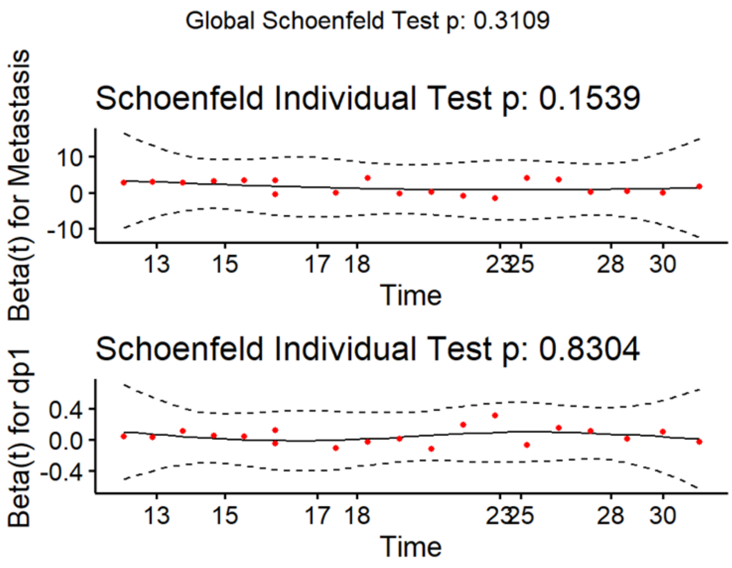


**b. OS-Model-2**

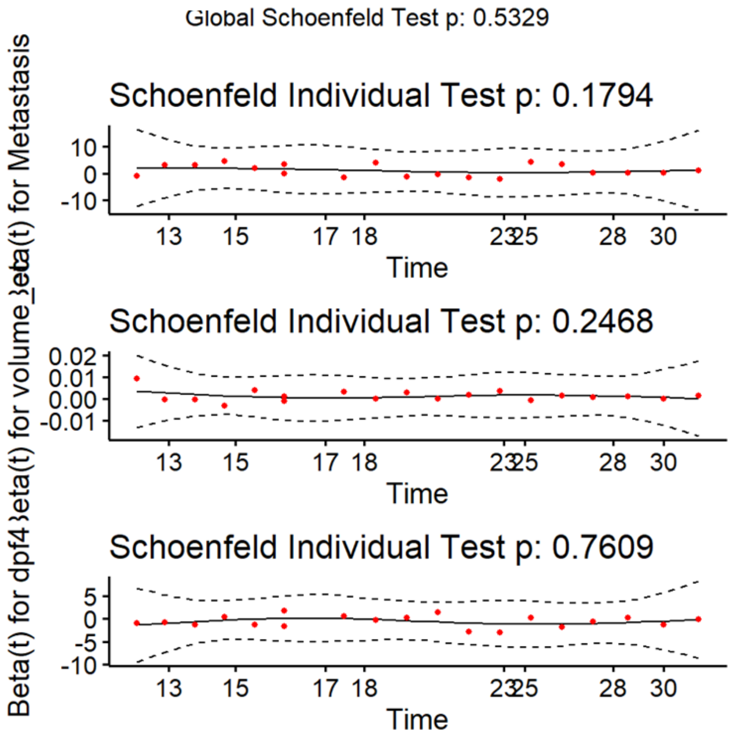

Supplement: Supplementary file 1 — Additional file 1. Additional Tables and figures. [file 12967_2022_3838_MOESM1_ESM.pdf]
